# FSH promotes immature porcine Sertoli cell proliferation by activating the CCR7/Ras-ERK signaling axis

**DOI:** 10.1530/REP-22-0441

**Published:** 2023-04-26

**Authors:** Yanfei Yin, Jiajia Ma, Xiaofang Lu, Saina Yan, Qianqian Jiang, Dazhi Wu, Bin Chen, Bo Weng, MaoLiang Ran

**Affiliations:** 1 College of Animal Science and Technology, Hunan Provincial Key Laboratory for Genetic Improvement of Domestic Animal, Hunan Agricultural University, Changsha, China; 2 Xiangxi Vocational and Technical College for Nationalities, Xiangxi, China; 3 Xiangtan Helong Ecological Agricultural Co., Ltd., Xiangtan, China

## Abstract

**In brief:**

The appropriate growth and functions of Sertoli cells are crucial to testis development and spermatogenesis in mammals. This study reveals a novel mechanism of follicle-stimulating hormone in immature porcine Sertoli cell proliferation.

**Abstract:**

Follicle-stimulating hormone (FSH) is a major Sertoli cell mitogen that binds to the FSH receptor. Sertoli cells are indispensable for testis development and spermatogenesis. However, the regulatory mechanisms of FSH in immature Sertoli cell proliferation have not been determined, particularly in domestic animals. In the present study, we identified the regulatory mechanisms of FSH during immature porcine Sertoli cell proliferation. Transcriptome analysis revealed 114 differentially expressed genes that were induced by FSH treatment, which contains 68 upregulated and 46 downregulated genes. These differentially expressed genes were enriched in multiple pathways, including the Ras signaling pathway. Knockdown of the CC-chemokine receptor 7 (*CCR7*) gene, which was upregulated by FSH, inhibited cell cycle progression by arresting cells in the G1 phase and reduced the cell proliferation and ERK1/2 phosphorylation. In addition, Kobe0065 inhibited Ras signaling in a similar manner as *CCR7* knockdown. Furthermore, FSH abolished the effects of Ras signaling pathway inhibition and *CCR7* knockdown. Collectively, FSH promotes immature porcine Sertoli cell proliferation by activating the CCR7/Ras-ERK signaling axis. Our results provide novel insights into the regulatory mechanism of FSH in porcine testis development and spermatogenesis by deciding the fate of immature porcine Sertoli cells.

## Introduction

Spermatogenesis is an extremely complicated and highly orchestrated biological process, which generates highly specialized mature spermatozoa from spermatogonial stem cells in the epithelium of the seminiferous tubules. In multiple spermatogenic cell types, the Sertoli cells are considered one of the most complex cell types based on their ability to continually modify shape and function in the processes of testis development and spermatogenesis (You *et al.* 2021, O'Donnell *et al.* 2022). During the fetal and prepubertal periods, immature Sertoli cells exhibit intense proliferation and promote testis development by driving cord lengthening and expansion, repressing pro-spermatogonia from entering meiosis, and promoting the differentiation of Leydig cells (Franca *et al.* 2016). During puberty, mature Sertoli cells enter a non-proliferation state, which guarantees generative cell development by establishing the blood–testis barrier, creating a suitable micro-environment, and secreting multiple nutrients and regulatory factors (Griswold 2018). Sertoli cell proliferation in pigs exhibits two distinct phases. The first occurs between birth and 1 month of age, in which the number of Sertoli cells per testis increases approximately 6-fold. The second occurs between 3 and 4 months of age or 4 and 5 months of age, in which Sertoli cells almost double their numbers per testis (Franca *et al.* 2000). However, the final number of Sertoli cells largely governs testis size and daily sperm production in adulthood as each mature Sertoli cell has a fixed capacity (Rebourcet *et al.* 2017). Therefore, the appropriate proliferation of Sertoli cells in an immature state is a key determinant for testis development and spermatogenesis.

Recently, existing literature has demonstrated that the proliferation of immature Sertoli cells is regulated by hormones (Nascimento *et al.* 2016), protein-coding genes (You *et al.* 2021), non-coding RNAs (Luo *et al.* 2020, Bian *et al.* 2021), and signaling pathways (Ni *et al.* 2019). Follicle-stimulating hormone (FSH) is a major Sertoli cell mitogen that binds to the FSH receptor. Knockout of the *FSHR* gene reduces the number of mouse Sertoli cells at birth by 22% and decreases the gametogenesis and daily sperm count in adulthood although it does not impair fertility (Abel *et al.* 2000). Furthermore, previous studies demonstrated that FSH controls immature Sertoli cell proliferation through multiple signal transduction pathways and/or genes *in vivo*. For example, FSH promotes the proliferation of rat immature Sertoli cells by activating the PI3K/Akt/mTORC1 pathway, whereas AMPK activation restrains the positive role of FSH by decreasing mTORC1 signaling and increasing *CDKI* expression (Riera *et al.* 2012). In addition, FSH reduces lysosomal biogenesis and further represses autophagy by activating the PI3K/Akt/mTOR pathway and upregulating the expression of *ABP*, *GDNF*, and *SCF* genes in dairy goat Sertoli cells (Xi *et al.* 2022). However, insight into the regulatory mechanisms of FSH in immature Sertoli cells is limited based on a large number of functional factors identified in this process (Mancuso *et al.* 2018), especially regarding immature porcine Sertoli cell proliferation.

To investigate the mechanisms of the action of FSH in immature porcine Sertoli cell proliferation, we used transcriptomics to examine the effect of FSH on gene expression. We identified an upregulated gene, CC-chemokine receptor 7 (*CCR7*) and the Ras-ERK signal transduction pathway that are associated with FSH activity based on previous investigations (Nascimento *et al.* 2012) and the analysis of our data. The results indicate that FSH regulates the expression of multiple genes and the activity of several signaling pathways. In addition, FSH promotes the proliferation of immature porcine Sertoli cells by upregulating *CCR7* expression and activating the Ras-ERK signaling pathway.

## Materials and methods

### Ethics statement

The present study was conducted according to the guidelines of the Declaration of Helsinki. All procedures involving animals were discussed and approved by the Ethics Committee of the Hunan Agricultural University (no. 2021-56). The animals did not suffer unnecessarily at any stage of the experiments.

### FSH content analysis

Shaziling boars (an indigenous pig breed from Hunan province, China) were provided by the Xiangtan Helong Ecological Agricultural Co. Ltd. (Xiangtan, China). A total of 21 boars were randomly divided into 1-day-old, 30-day-old, 60-day-old, 90-day-old, 120-day-old, 150-day-old, and 180-day-old groups. Each group was individually housed in a pen (10 m^2^) and fed and managed according to the standards for Shaziling pigs (DB43/T 625-2011). The boars were hand-fed three times/day (7:30, 11:30, and 17:30 h) in feeding troughs to ensure that fresh feed was available and* ad libitum*. Water was also provided* ad libitum*. Testicular tissue samples were collected by castration under sterile conditions and general anesthetic (Zoletil 50, Virbac Co., Nice, France). The fresh testicular tissue samples were washed three times with pre-chilled phosphate-buffered saline (PBS, pH 7.4). Then, a 50 mg testicular tissue sample was homogenized with 450 μL PBS. FSH content of the testis tissue homogenates was further determined using a porcine FSH ELISA kit (FANKEW Co., Shanghai, China, no. F4567-A) with an ELISA plate reader (Molecular Devices, San Francisco, CA, USA) at 450 nm.

### Cell isolation, culture, and transfection

Sertoli cells were isolated from the testis tissues of 21-day-old Shaziling boars based on previously described methods (Yang *et al.* 2020, Deng *et al.* 2022). Briefly, the fresh testis samples were washed three times with pre-chilled PBS (pH 7.4) after removing the epididymis and fatty tissue. The tissues were sheared into fragments and incubated in PBS (pH 7.4). After centrifugation at 1000 ***g*** for 5 min, the pellets were digested with 0.3% type-V collagenase and 0.25% trypsin at 32°C for 25 min. The products were filtered sequentially through 70 and 100 μm mesh to obtain a Sertoli cell suspension. The Sertoli cells were validated using RT-PCR and fluorescence *in situ* hybridization assays for their characteristic marker genes (Supplementary Fig. 1, see section on supplementary materials given at the end of this article). The Sertoli cells were cultured in Dulbecco's modified Eagle medium (HyClone, Logan, UT, USA) containing 10% fetal bovine serum (Gibco) at 32°C with 5% of CO_2_.

When the cells reached approximately 80% confluence, functional gain or loss assays were performed. For treatment, FSH was diluted in ddH_2_O and added to the cell medium at 0, 25, 50, 75, 100, 125, 150, 175, or 200 ng/mL. For *CCR7* knockdown, 100 pmol (final concentration, 50 nM in the cells) *CCR7* siRNA (GenePharma, Suzhou, Jiangsu, China) (Supplementary Table 1) or siRNA NC (GenePharma) was diluted with 250 μL of serum-free Opti-MEM and transfected with Lipofectamine^TM^ 2000 (Invitrogen). To inhibit Ras signaling, Kobe0065 (the specific inhibiter of Ras signaling pathway, ThermoFisher, no. B3586) was diluted in DMSO (final concentration, 0.1% in the cells) and added to the medium at a final concentration of 10 μM. After incubating for 6–8 h at 32°C with 5% of CO_2_, the above-mentioned complete medium was used for subsequent cell culture.

### Transcriptome analysis

Immature porcine Sertoli cells were treated with FSH (75 ng/mL) or ddH_2_O for 72 h. The cells were collected and total RNA was extracted using TRIzol reagent (Invitrogen). RNA integrity was assessed using the RNA Nano 6000 Assay Kit and the Bioanalyzer 2100 system (Agilent Technologies). Only the samples with RNA integrity number scores higher than 8 were used for subsequent experiments. A total of six cDNA libraries were conducted using the NEBNext Ultra Directional RNA Library Prep Kit for Illumina. These prepared libraries were sequenced on an Illumina HiSeq 4000 platform provided by the Shanghai Personalbio Technology Co., Ltd. (Shanghai, China). Differentially expressed genes were identified according to a threshold of *P*-adjust < 0.05 and |log_2_(fold-change)|>1. Gene Ontology (GO) enrichment analysis was performed using the GOseq R package and Kyoto Encyclopedia of Genes and Genomes (KEGG) enrichment analysis was performed on KOBAS software as described previously (Ran *et al.* 2015, 2016, Weng *et al.* 2017). The raw sequence data reported in this paper have been deposited in the Genome Sequence Archive in National Genomics Data Center, China National Center for Bioinformation/Beijing Institute of Genomics, Chinese Academy of Sciences (GSA: CRA010109) that are publicly accessible at https://ngdc.cncb.ac.cn/gsa.

### Cell cycle assay

After a 72 h transfection, the cells were washed three times with PBS (pH 7.4) and harvested into a 1.5 mL centrifuge tube. The cells were incubated in 70% (v/v) ethanol for 12 h at −20°C followed by incubation with propidium iodide solution (50 mg/mL) for 30 min at 4°C. The cell suspension was analyzed using a cell cycle testing kit (Nanjing KeyGen Biotech, Nanjing, Jiangsu, China) on a FACScanto II flow cytometer (Becton Dickinson, Franklin Lake, NJ, USA).

### CCK-8 assay

The cell counting kit-8 (CCK-8) assay was used as described previously (Gao *et al.* 2019, Luo *et al.* 2020). The cells were seeded into a 96-well culture plate at a density of 1 × 10^4^ cells/well in 100 μL of culture medium. Then, 10 μL of CCK-8 medium (Multiscience, Hangzhou, Zhejiang, China) was added to each well and the plate was incubated at 32°C for 4 h. The absorbance value of each well was measured using an ELISA plate reader (Molecular Devices) at 450 nm.

### EdU assay

Cells were seeded in a 96-well culture plate at a density of 1 × 10^4^ cells/well in 100 μL of culture medium. Then, 100 μL 5-ethynyl-2´-deoxyuridine (EdU) medium (50 μmol, Ribobio, Guangzhou, Guangdong, China) was added to each well and the plate was incubated at 32°C for 2 h. DNA and EdU staining solution were subsequently added to each well to identify living cells (blue) and proliferating cells (red) based on the manufacturer’s protocol, respectively. Finally, the cells were observed under a fluorescence microscope (ThermoFisher).

### ATP assay

Cells were seeded into a 96-well culture plate at a density of 1 × 10^4^ cells/well in 100 μL of culture medium. The adenosine triphosphate (ATP) concentration was measured using an ATP assay kit (Beyotime, Shanghai, China) based on the manufacturer’s protocol. The relative ATP levels of the experimental groups were normalized to that of the negative control group.

### Real-time PCR

Total RNA was extracted using TRIzol reagent (Invitrogen) based on the manufacturer’s protocol. The RNA yield and quality were checked using a nucleic acid ultramicro detector (ThermoFisher) (Supplementary Table 1). Primer sequences were designed using Oligo 7.0 software (Supplementary Table 2) and synthesized by Sango Bio. (Shanghai, China). Complementary DNA (cDNA) was prepared from each sample using the PrimeScript first-strand cDNA synthesis kit (TaKaRa) according to the manufacturer's protocol. Then, an RT-PCR assay was conducted to validate the primer specificity, cDNA quality, and annealing temperature (Supplementary Fig. 2). Quantitative PCR was done using a Thermo Scientific PIKO REAL 96 real-time PCR System with an SYBR Green kit (TaKaRa). Each qRT-PCR reaction mixture (25 μL) reaction contained 2.0 μL cDNA, 1 μL sense primer (10 μM), 1 μL antisense primer (10 μM), 8.5 μL ddH_2_O, and 12.5 μL SYBR Premix Ex Taq II (TaKaRa). The reaction conditions were 95°C for 10 min, followed by 40 cycles of 95°C for 10 s, and 60±4°C (Supplementary Table 2) for 50 s. All qRT-PCR reactions were performed in triplicate. The melt curve of each gene was provided in Supplementary Fig. 2. The *β-actin* gene was used as an internal control. The relative expression of each gene was determined using the 2^−△△Ct^ method.

### Western blot analysis

Total cell protein was extracted using radioimmunoprecipitation assay lysis buffer (Beyotime). Protein concentration was measured using the bicinchoninic acid protein assay kit (Beyotime) according to the manufacturer’s protocols. The protein samples were boiled, electrophoresed on 10% SDS-polyacrylamide gels, and transferred to a PVDF membrane (Beyotime). The membrane was blocked with 5% non-fat milk for 2 h and incubated with primary antibodies at 4°C for 12 h. The antibodies included CCR7 (1:4000, HUA Bio, Chengdu, Sichuan, China, no. ET1602-22), ERK1/2 (1:1000, Cell Signaling Technology, no. AF0155), p-ERK1/2 (1:5000, Proteintech Group, Chicago, IL, USA, no. 4370T), and β-actin (1:5000, Proteintech Group, no. Biotin-60008). After washing, the membrane was incubated with a secondary antibody (1:5000, Proteintech Group, no. A0208, no. A0216) for 2 h at 26°C. Protein bands were visualized using an ECL advanced western blot detection kit (Beyotime). β-actin served as the loading control. Each band was quantified using ImageJ software (National Institutes of Health). Then, the target protein/β-actin ratio was calculated to quantify these western blot results.

### Statistical analysis

Data are presented as the mean ± s.d. All experiments were performed in triplicate. Data from multiple cell groups were subjected to a one-way ANOVA followed by Duncan’s multiple comparisons test of significance using SPSS 17.0 software (IBM). A *t*-test was used to test the differences in data from only two cell groups. *P* < 0.05 or *P* < 0.01 was considered statistically significant.

## Results

### FSH promotes immature porcine Sertoli cell proliferation

**Figure 1:**
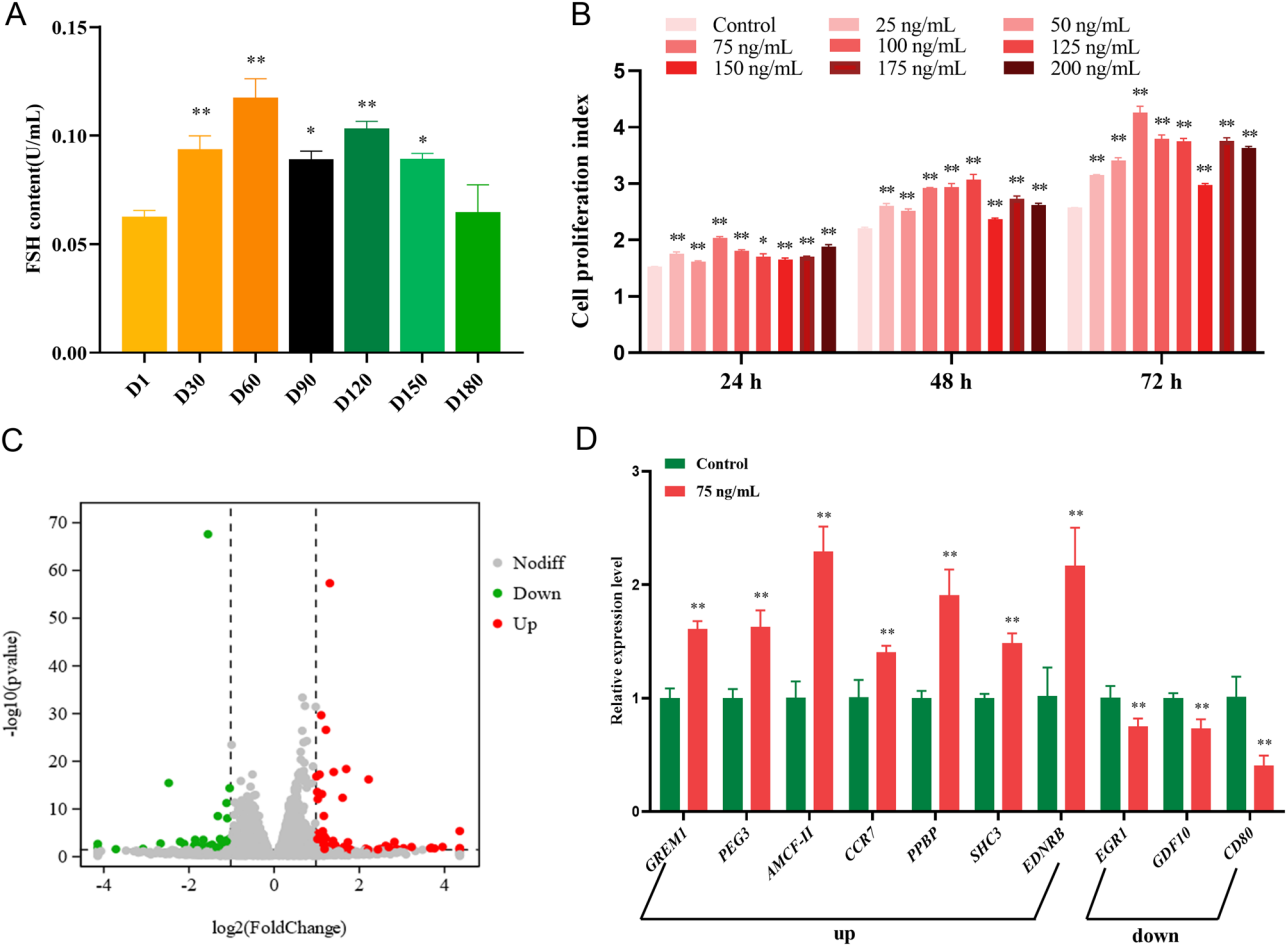
A transcriptome approach characterizes the effects of gene expression in response to the FSH. (A) The FSH content in developing postnatal Shaziling boar testicular tissues including 1-day-old, 30-day-old, 60-day-old, 90-day-old, 120-day-old, 150-day-old, and 180-day-old. (B) The effect of FSH on immature porcine Sertoli cell proliferation was measured using the CCK-8 assay, and the ddH_2_O was used as the negative control. (C) The immature porcine Sertoli cells were treated with ddH_2_O or FSH (75 ng/mL) for 72 h, and these six libraries were sequenced on an Illumina HiSeq 4000 platform. A total of 114 differentially expressed genes were identified with 68 being upregulated and 46 downregulated using a *P* < 0.05 and |log_2_(fold change)|>1 as the cutoff. (D) The differentially expressed genes were validated using the qRT-PCR assay. The β-actin gene was used as the internal control. All experiments were constructed with at least three replicates. Data were presented as the mean ± s.d. *P < 0.05 and **P < 0.01.

To determine the relationship between FSH content and porcine Sertoli cell proliferation, FSH content was measured during postnatal boar testis development, which included seven developmental time points (Fig. 1A). The results indicated that FSH content significantly increased from 1-day-old to 60-day-old boars and markedly decreased from 90-day-old to 180-day-old boars (Fig. 1A). In addition, the results indicated that immature porcine Sertoli cell proliferation activity was significantly increased by eight FSH treatments (*P* < 0.05) and the immature porcine Sertoli cells exhibited highest proliferation activity following treatment with 75 ng/mL FSH for 72 h (*P* < 0.01) (Fig. 1B). The results indicate that FSH contributes to the regulation of immature porcine Sertoli cell proliferation.

### Transcriptome analysis of the regulatory role of FSH

**Figure 2:**
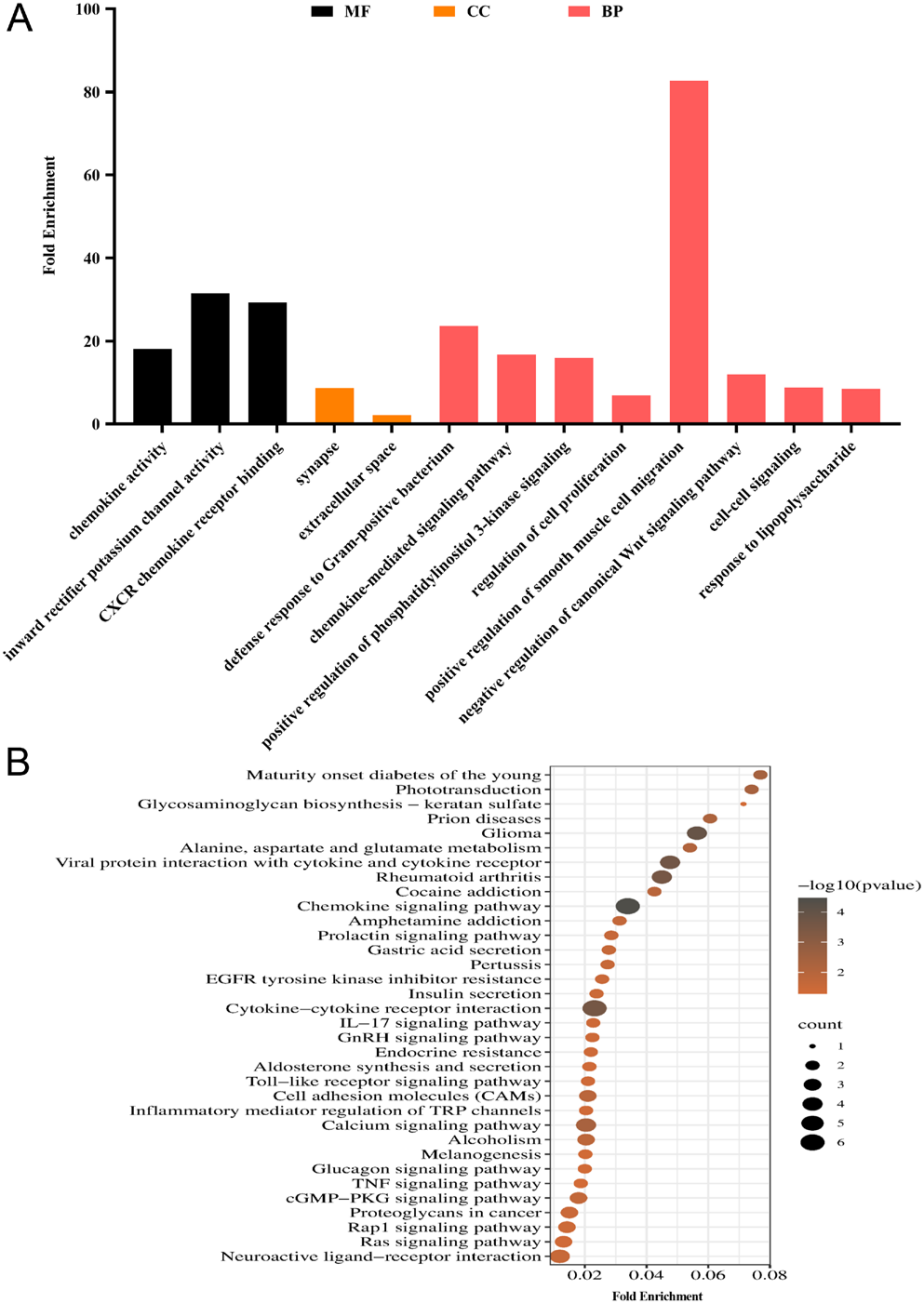
The functional enrichment results of differentially expressed genes. (A) The GO enrichment results of differentially expressed genes. BP, biological process; CC, cellular component; MF, molecular function. (B) The KEGG enrichment results of differentially expressed genes.

To determine the regulatory role of FSH on gene expression in immature porcine Sertoli cells, a transcriptomic approach was applied to immature porcine Sertoli cells treated with NC (ddH_2_O) or FSH. A total of 281,962,412 clean reads were obtained from six libraries. Using a *P*-adjust < 0.05 and |log_2_(fold-change)|>1 as a cutoff, a total of 114 differentially expressed genes were identified, 68 upregulated and 46 downregulated (Fig. 1C and Supplementary Table 3). In addition, the relative mRNA levels of 10 randomly selected differentially expressed genes were validated by qRT-PCR (Fig. 1D). The differentially expressed genes were analyzed using the GO and KEGG pathway databases. GO enrichment analysis revealed a total of 13 GO terms that were significantly enriched and several of these may explain the regulatory role of FSH on immature porcine Sertoli cell proliferation (Fig. 2A), including positive regulation of phosphatidylinositol 3-kinase signaling (GO: 0090090), negative regulation of canonical Wnt signaling pathway (GO: 0090090), and regulation of cell proliferation (GO: 0042127). Furthermore, KEGG pathway enrichment analysis revealed that these differentially expressed genes were significantly enriched in 34 pathways (Fig. 2B), including Ras (ssc04014), Rap1 (ssc04668), and TNF (ssc04015) signaling pathways.

### CCR7 knockdown inhibits immature porcine Sertoli cell proliferation

**Figure 3:**
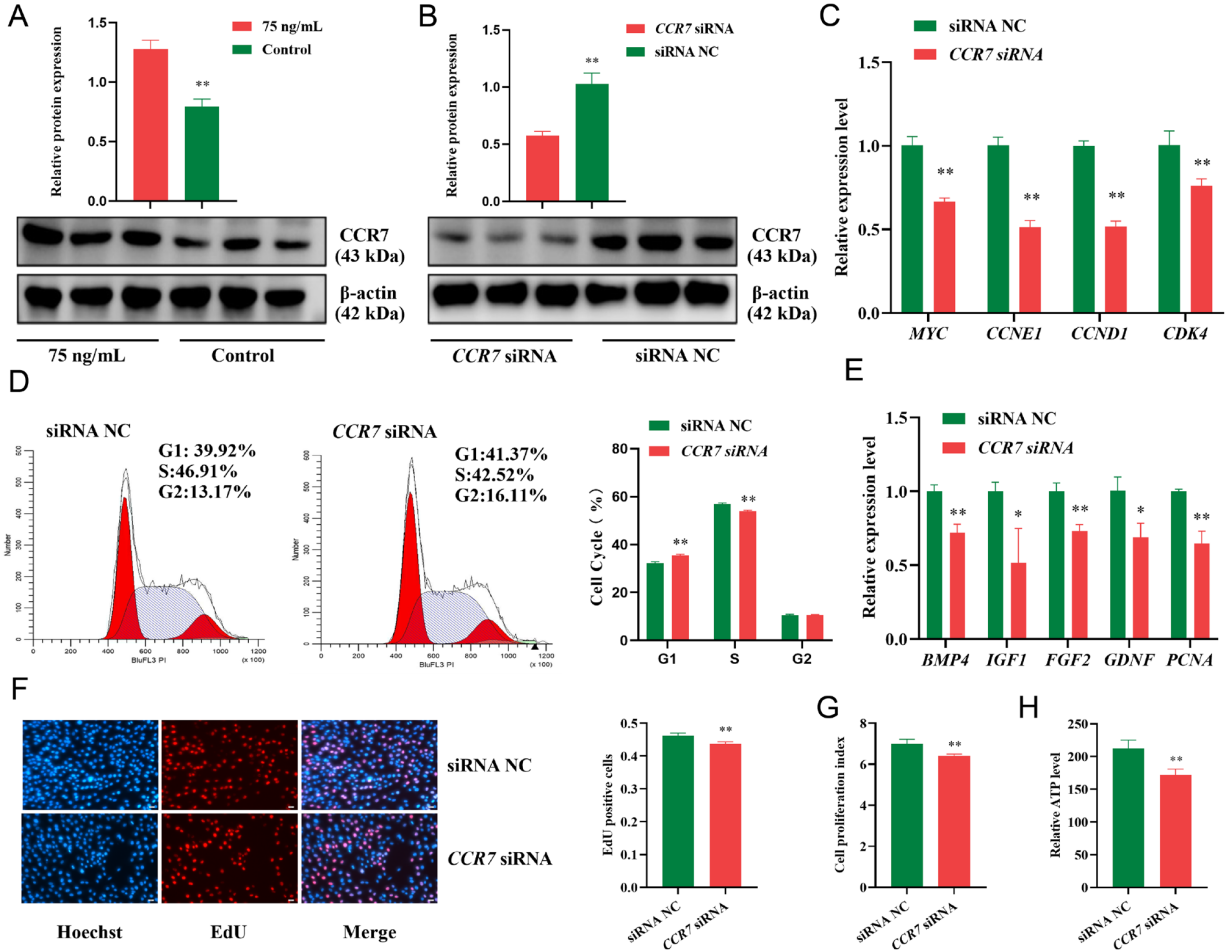
Knockdown of *CCR7* inhibits immature porcine Sertoli cell proliferation. (A) FSH (75 ng/mL) increased the CCR7 protein expression. The ddH_2_O was used as the negative control. (B) A specific siRNA induced the knockdown of CCR7 immature porcine Sertoli cells. (C) The mRNA expression of cell cycle-related genes was accessed using the qRT-PCR assay. The β-actin gene was used as the internal control. (D) The cell cycle distribution was analyzed using a FACSCanto II Flow Cytometer. (E) The mRNA expression of cell proliferation-related genes was accessed using the qRT-PCR assay. The β-actin gene was used as the internal control. (F, G, H) The cell proliferation induced by the FSH was determined using the EdU incorporation (F), CCK-8 (G), and ATP (H) assays. All experiments were constructed with at least three replicates. Data were presented as the mean ± s.d. *P < 0.05 and **P < 0.01.

*CCR7* was selected as a candidate gene to further explore the potential mechanism of FSH in immature porcine Sertoli cell proliferation by the western blot analysis (*P* < 0.01) (Fig. 3A). A specific siRNA for the *CCR7* gene was transfected into immature porcine Sertoli cells to knockdown its expression (*P* < 0.01) (Fig. 3B). Cell cycle analysis revealed that *CCR7* inhibition significantly elevated the percentage of cells in the G1 phase (*P* < 0.01) and significantly decreased the S phase cell population compared with that of the NC group (*P* < 0.01) (Fig. 3D). In addition, the relative mRNA expression of cell cycle-related genes was significantly downregulated by *CCR7* knockdown (*P* < 0.01), including *MYC, CCNE1*, *CCND1*, and *CDK4* (Fig. 3C). Furthermore, siRNA treatment caused a significant reduction in *CCR7* and concomitantly decreased the relative mRNA expression of cell proliferation-related genes (*P* < 0.05), including *BMP4*, *IGF1*, *FGF2*, *GDNF*, and *PCNA* (Fig. 3E). Similarly, the results from the CCK-8 and EdU incorporation assays revealed that the cell proliferation index and mitotic activity were both inhibited by *CCR7* knockdown (*P* < 0.01), respectively (Fig. 3F and G). Relative ATP levels were also decreased by siRNA-mediated *CCK7* inhibition (*P* < 0.01) (Fig. 3H). Collectively, these results indicate that *CCR7* knockdown restrains cell cycle progression and cell proliferation.

### CCR7 inhibition antagonizes the effects of FSH

**Figure 4:**
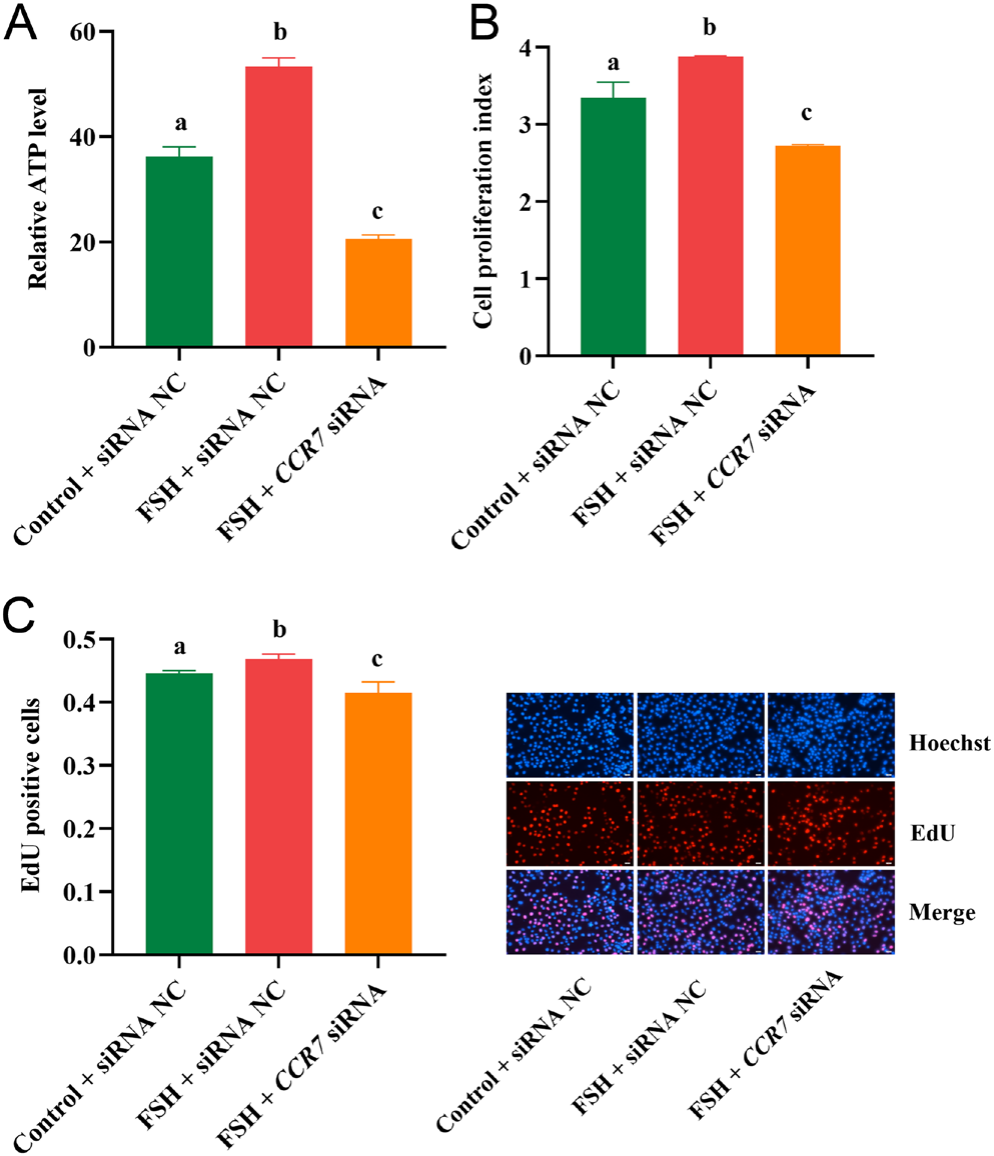
Knockdown of *CCR7* abolishes the effects of FSH. A total of three co-treatments were constructed, including control (ddH_2_O) + siRNA NC, FSH (75 ng/mL) + siRNA NC, FSH (75 ng/mL) + *CCR7* siRNA. ATP (A), CCK-8 (B), and EdU incorporation (C) assays were used to investigate cell proliferation. All experiments were constructed with at least three replicates. Data were presented as the mean ± s.d.

To further confirm that the *CCR7* gene is involved in the signaling axis of FSH during the regulation of immature porcine Sertoli cell proliferation, the cells were treated with control + siRNA NC, FSH + siRNA NC, and FSH + *CCR7* siRNA. The results indicated that FSH exposure resulted in a higher relative ATP level (*P* < 0.01), whereas this effect was abolished by *CCR7* inhibition (*P* < 0.01) (Fig. 4A). Consistently, the results from CCK-8 and EdU incorporation assays demonstrated that* CCR7* knockdown attenuated the FSH-induced higher cell proliferation activity (*P* < 0.05) (Fig. 4B and C). These results indicate that *CCR7* inhibition antagonizes the effects of FSH on immature porcine Sertoli cell proliferation.

### The Ras signaling pathway participates in the mechanism of the action of FSH

**Figure 5:**
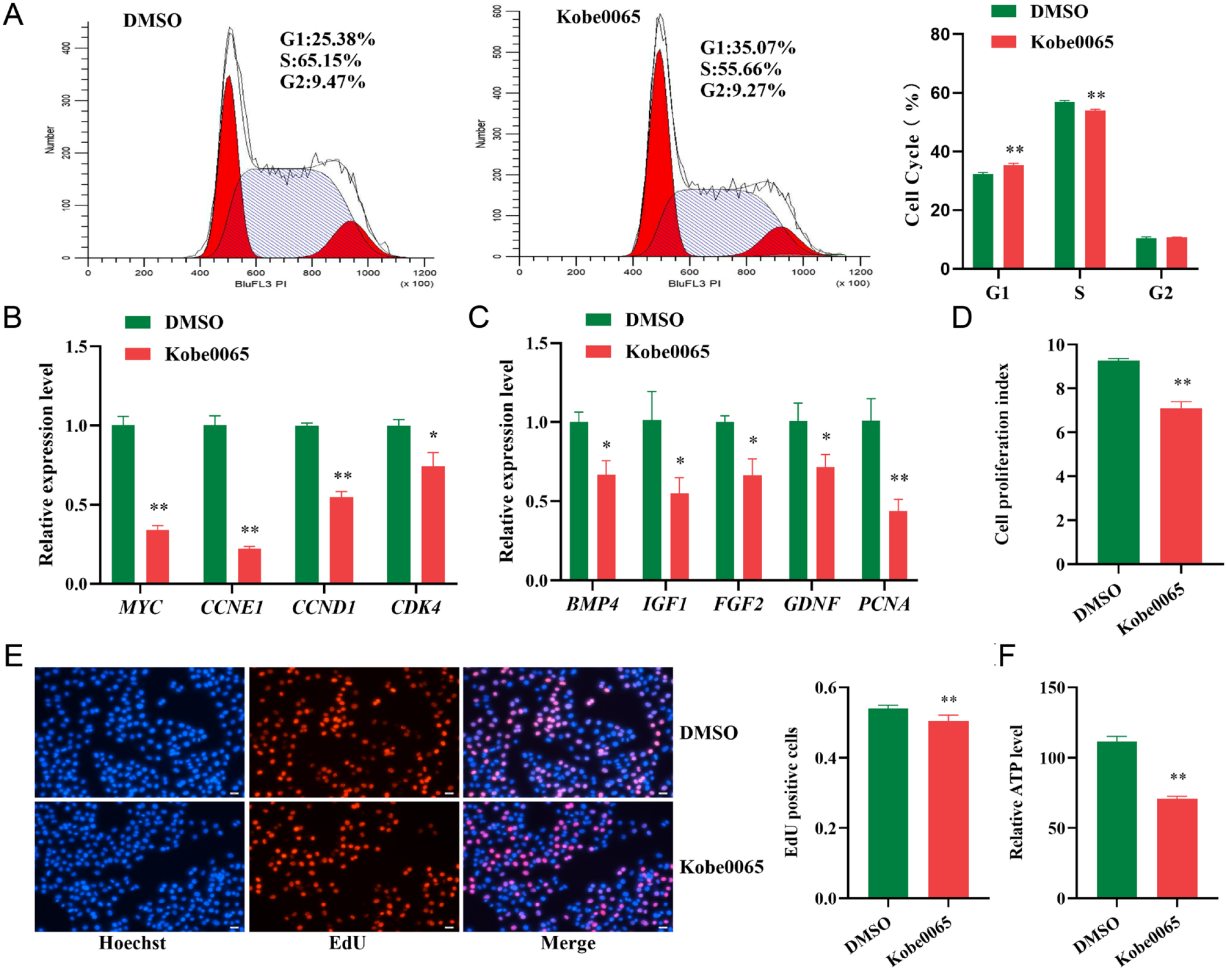
Inhibition of the Ras singing pathway represses immature porcine Sertoli cell proliferation. The DMSO and Kobe0065 were added in the culture medium of immature porcine Sertoli cells to function as NC and the inactivation of the Ras singing pathway, respectively. (A) The cell cycle distribution was analyzed and then the cell proportion of the G1, S, and G2 phases was counted. (B, C) The mRNA expression of (B) cell cycle- and (C) cell proliferation-related genes were accessed using the qRT-PCR assay. The β-actin gene was used as the internal control. (D, E, F) CCK-8 (D), EdU incorporation (E), and ATP (F) assays were used to investigate cell proliferation. All experiments were constructed with at least three replicates. Data were presented as the mean ± s.d. *P < 0.05 and **P < 0.01.

**Figure 6:**
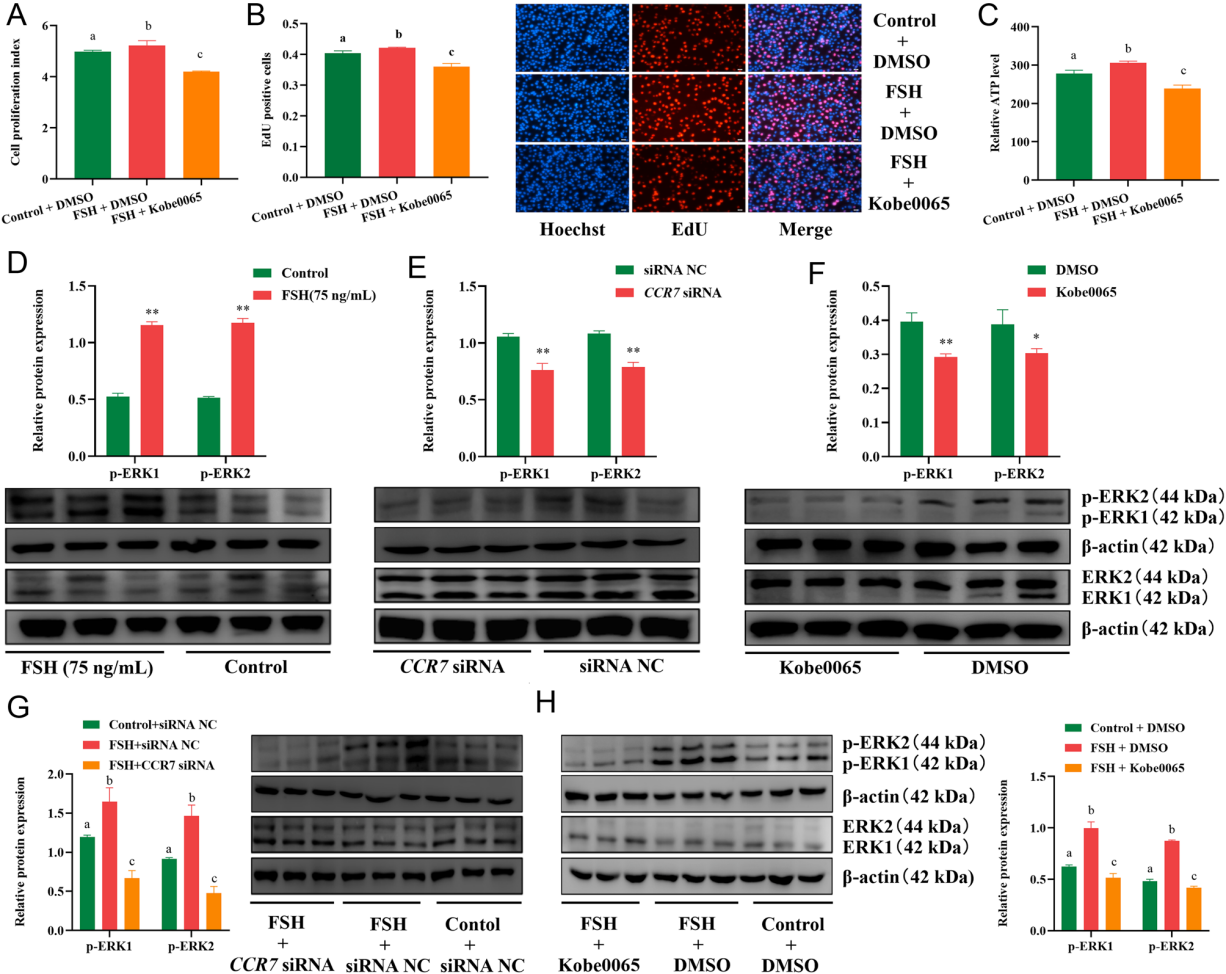
Inhibition of the Ras singing pathway offset the effects of FSH. A total of three co-treatments were constructed, including control (ddH_2_O) + DMSO, FSH (75 ng/mL) + DMSO, FSH (75 ng/mL) + Kobe0065. (A, B, C) CCK-8 (A), EdU incorporation (B), and ATP (C) assays were used to investigate cell proliferation. (D, E, F, G, H) The ERK1/2 phosphorylation was measured in immature porcine Sertoli cells treated with FSH (75 ng/mL) (D), *CCR7* siRNA (E), Kobe0065 (F), FSH (75 ng/mL) + *CCR7* siRNA (G), FSH (75 ng/mL) + Kobe0065 (H) using the western blot assay. The β-actin was used as the internal control. All experiments were constructed with at least three replicates. Data were presented as the mean ± s.d. *P < 0.05 and **P < 0.01. Different letters indicate mean values within each section were significantly different.

The results of KEGG enrichment analysis indicated that the FSH-induced differentially expressed genes were significantly associated with the Ras signaling pathway (Fig. 2B). Therefore, a specific inhibitor of the Ras signaling pathway (Kobe0065) was added to the cell culture medium to inhibit its activity. Cell cycle analysis revealed that the inactivation of Ras signaling induced more cells in the G1 phase and fewer cells in the S phase (*P* < 0.01) (Fig. 5A). Similarly, the relative mRNA expression of cell cycle-related genes was significantly decreased by Ras signaling inhibition (*P* < 0.05) (Fig. 5B). In addition, immature porcine Sertoli cell proliferation was significantly reduced by inhibiting Ras signaling (*P* < 0.01) as evidenced by the results from qRT-PCR (Fig. 5C), CCK-8 (Fig. 5D), and EdU (Fig. 5E) incorporation assays. Consistently, the relative ATP levels in immature porcine Sertoli cells were decreased by Ras signaling pathway inhibition (*P* < 0.01) (Fig. 5F). Next, cells were then co-treated with FSH and Kobe0065 to determine whether the Ras signaling pathway participates in the mechanism of action of FSH. The results indicated that inhibition of Ras signaling attenuated the FSH-induced higher cell proliferation index (Fig. 6A) and mitotic activity (Fig. 6B). Furthermore, the higher relative ATP levels induced by FSH were also abolished by inhibition of the Ras signaling pathway (Fig. 6C). These results indicate that Ras signaling pathway participates in the regulatory mechanism of FSH on immature porcine Sertoli cell proliferation.

### FSH activates the ERK signaling through the elevation of CCR7 and activation of the Ras signaling pathway

Ras/Raf/MEK/ERK signaling is a classic pathway that regulates cell proliferation. Therefore, we further determined whether FSH signaling is transmitted from Ras to ERK. The results indicated that FSH significantly increased ERK1/2 phosphorylation (*P* < 0.01) (Fig. 6D), as well as both inhibition of *CCR7* and Ras signaling decreased ERK1/2 phosphorylation(*P* < 0.05) (Fig. 6E and F), whereas the effect of FSH on ERK1/2 protein was abolished by *CCR7* knockdown of or Ras signaling pathway inhibition (*P* < 0.05) (Fig. 6G and H). These data indicated that FSH activates the ERK signaling by upregulating the *CCR7* expression and Ras signaling activity.

## Discussion

**Figure 7:**
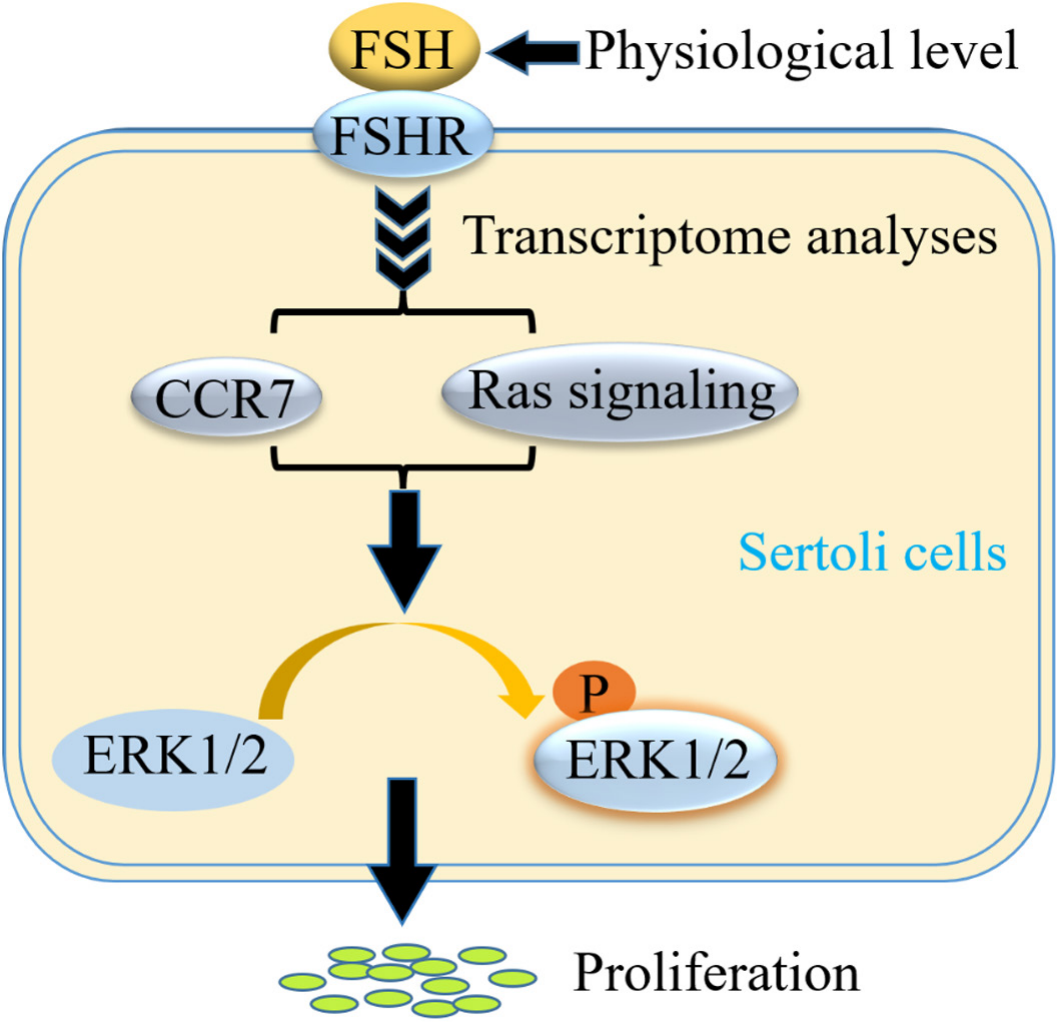
Model of the main investigations of this study. FSH promoted immature porcine Sertoli cell proliferation by activating the CCR7/Ras-ERK signaling axis.

Decades of *in vivo* and *in vitro* studies have revealed that Sertoli cells are the primary targets of FSH and FSH signaling is required for Sertoli cell proliferation during fetal and early postnatal life. FSH binds to the FSH receptor, which is exclusively expressed on the surface of Sertoli cells, and stimulates downstream mRNA-specific translation and signaling, such as the cAMP/PAK, ERK/MAPK, and PI3K signaling pathways (Wang *et al.* 2022). However, the regulatory mechanisms associated with FSH in immature porcine Sertoli cell proliferation remain unclear as studies have focused more on female fertility. In the present study, a transcriptomic approach was conducted to investigate the transcriptional regulatory roles of FSH. We also determined that FSH promotes immature porcine Sertoli cell proliferation by upregulating *CCR7* expression and activating the Ras-ERK signaling pathway (Fig. 7).

Using RNA sequencing, a total of 114 differentially expressed genes were identified with 68 upregulated and 46 downregulated. Several of these differentially expressed genes participated in FSH signaling to control immature porcine Sertoli cell proliferation. For example, the insulin-like growth factor 1 (IGF1), which was upregulated, is involved in FSH-modulated porcine Sertoli cell proliferation and function through a signaling axis with growth hormone (Cannarella *et al.* 2019). In addition, the IGF1 receptor (IGF1R) has important roles in FSH signaling, which is supported by the finding that the IGF1R inhibitor NVP-AEW541 inhibits FSH-induced MYPT1 and ERK1/2 phosphorylation, decreases FSH-dependent protein kinase B (AKT)^308^ phosphorylation, and inhibits the FSH-induced *AMH* and *FSHR* downregulation (Cannarella *et al.* 2019). Furthermore, the results of an enrichment analysis revealed that these abovementioned differentially expressed genes were enriched in multiple signaling pathways, including Ras, cGMP-PKG, TNF, and TLR signaling pathways. The activation of the cGMP-PKG signaling pathway mediates the positive regulatory effect of C-type natriuretic peptide on the mRNA expression of androgen-binding protein and transferrin in the Sertoli cells (Yu *et al.* 2021). TNF signaling enriched pathway identified in the present study, mediates *SPATA2* gene-induced cell death by recruiting CYLD to the TNFR1 signaling complex (Masola *et al.* 2022). In addition, several TLRs are expressed in Sertoli cells and can initiate an innate immune response to pathogens after ligand activation, including TLR2, TLR3, and TLR4 (Hedger 2011, Zhang *et al.* 2013, Di Pietro *et al.* 2020). Taken together, the regulatory mechanisms of FSH on the proliferation and function of immature porcine Sertoli cells involve multiple genes and signaling pathways. A proteomic or metabonomic approach needs to be conducted to further explore the regulatory mechanisms of FSH.

In the present study, the *CCR7* gene was identified as a potential regulatory target of FSH in immature porcine Sertoli cells based on the transcriptome, qRT-PCR, and western blot analysis. The *CCR7* gene regulates biological processes in many cell types through various signaling pathways. For example, CCR7 enhances human endometrial stromal cell proliferation by activating the PI3K/Akt signaling pathway (Diao *et al.* 2017), prevents non-small cell lung cancer apoptosis through the NF-κB and ERK signaling pathways (Xu *et al.* 2012, Zhang *et al.* 2017), and promotes the migration and invasion of urinary bladder cancer cells via the MEK/ERK1/2 signaling pathway (Xiong *et al.* 2017). *In vitro*, *Ccr7*-deficient mice exhibit less mesangial cell proliferation between embryonic day E17.5 and week 5 postpartum (Wurm *et al.* 2018). In addition, the *CCR7* gene also contributes to G2/M phase progression by upregulating cyclin A, cyclin B1, and CDK1, possibly through the ERK pathway (Xu *et al.* 2011, Zhang *et al.* 2016). However, to our knowledge, there has been no basis to examine the regulatory roles of the *CCR7* gene on immature porcine Sertoli cell proliferation. In the present study, siRNA-induced *CCR7* knockdown arrested cells in the G1 phase, inhibited immature porcine Sertoli cell proliferation, and reduced the phosphorylation of ERK1/2. Furthermore, *CCR7* knockdown further antagonized the effects induced by FSH administration. Our results together with other investigations indicate that FSH promotes immature porcine Sertoli cell proliferation through the *CCR7*-mediated activation of the ERK signaling pathway.

The enrichment results also revealed that these differentially expressed genes were significantly enriched in the Ras signaling pathway, which is known to regulate cell proliferation. Ras can transmit regulatory signals to multiple pathways, including ERK, PI3K/Akt, MAPK, and NF-κB (Liu *et al.* 2021, Pan *et al.* 2021, Guo *et al.* 2022). In addition, the Ras/RAF/MEK/MAPK signaling pathway participates in the regulation of proliferation in multiple cell types (Chang *et al.* 2003). Based on these investigations, we used the Ras/RAF specific inhibitor, Kobe0056, to treat immature porcine Sertoli cells. The results demonstrated that Kobe0056 arrested cells in the G1 phase, reduced cell proliferation, decreased ERK1/2 phosphorylation, and abolished the aforementioned effects induced by FSH. Previous studies indicate that the activation of ERK signaling is necessary for regulating multiple biological processes in Sertoli cells, including cell proliferation (Xu *et al.* 2020, Zhang *et al.* 2020), apoptosis (Choi *et al.* 2014, Zhang *et al.* 2020), secretion (Gomez *et al.* 2012), and the integrity of the blood–testis barrier (Zhang *et al.* 2014). Interestingly, both *CCK7* and Ras mediated the positive effects of FSH on the ERK signaling pathway to regulate the immature porcine Sertoli cell proliferation (Fig. 7).

## Conclusion

In summary, we examined the effects of FSH on the transcriptome of immature porcine Sertoli cells using RNA sequencing, which provided a basis for further examining the regulatory mechanisms of FSH in immature porcine Sertoli cell proliferation. Our results indicate that FSH promotes immature porcine Sertoli cell proliferation through the activation of the CCR7/Ras-ERK signaling axis. We hypothesize that these FSH signaling-related molecules exhibit important regulatory roles in porcine testis development and spermatogenesis and contribute to various biological processes in Sertoli cells.

## Supplementary Material

Figure S1 The validation of immature porcine Sertoli cells. (A) The marker genes of immature Sertoli cells were determined using the RT-PCR assay. (B) Immunofluorescence staining for the Sertoli cell marker SOX9 (green) and DAPI (blue).

Figure S2 The validation of primer specificity, cDNA quality, and annealing temperature.

Figure S3 The melt curve of each gene in the qRT-PCR assay.

Table S1 The RNA yield and quality in the present study.

Table S2 The sequences of primer and siRNA used in the present study.

Table S3 The differentially expressed genes induced by FSH.

## Declaration of interest

The authors declare no conflict of interest.

## Funding

This study was supported by special funds for Changsha Municipal Natural Science Foundation (kq2202229), Hunan Provincial Natural Science Foundation of China (2020JJ4348), and a key R&D projects in Hunan Province (2020NK2024).

## Data availability statement

The data supporting the results of this study can be obtained from the corresponding author according to reasonable request.

## Author contribution statement

MR and BW contributed to the experimental conception and design. FY, JM, XL, SY, QJ, DW, and BC performed the experiments. FY and MR analyzed the data and wrote the first draft of the manuscript. All authors reviewed and approved the final manuscript.
