# Supplementary figures and images for: FSH promotes immature porcine Sertoli cell proliferation by activating the CCR7/Ras-ERK signaling axis
Source: Reproduction. 2023 Apr 26;165(6):593–603. doi: 10.1530/REP-22-0441 (PMC10235919; doi:10.1530/REP-22-0441)

A

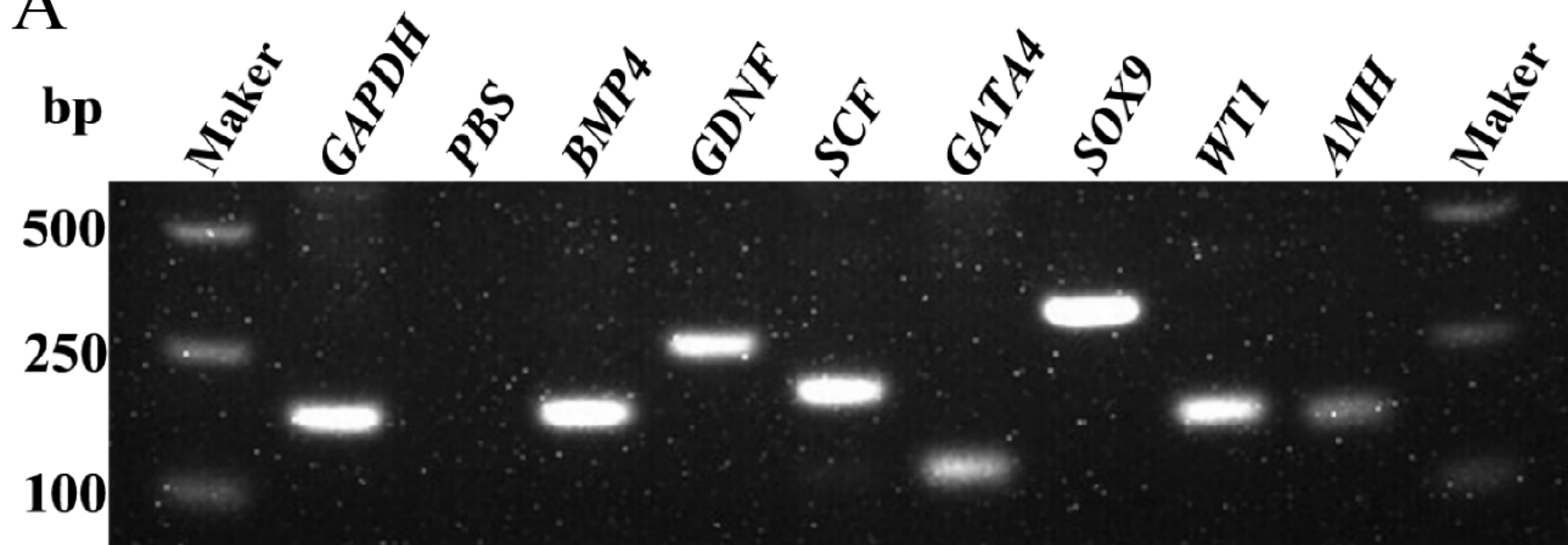

B

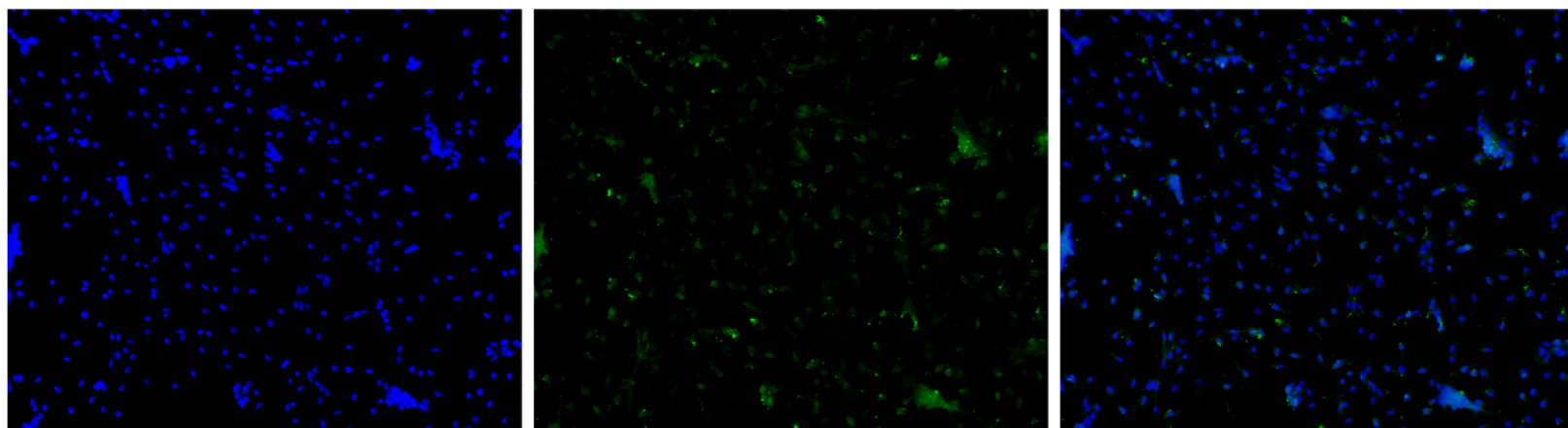

DAPI

SOX9

Merge

Supplement: Figure S1 The validation of immature porcine Sertoli cells. (A) The marker genes of immature Sertoli cells were determined using the RT-PCR assay. (B) Immunofluorescence staining for the Sertoli cell marker SOX9 (green) and DAPI (blue). [file supplementary_figure_1.pdf]
