# Supplementary material for: FSH promotes immature porcine Sertoli cell proliferation by activating the CCR7/Ras-ERK signaling axis
Source: Reproduction. 2023 Apr 26;165(6):593–603. doi: 10.1530/REP-22-0441 (PMC10235919; doi:10.1530/REP-22-0441)
Supplement: Figure S2 The validation of primer specificity, cDNA quality, and annealing temperature. [file supplementary_figure_2.pdf]

FSH promotes immature porcine Sertoli cell proliferation by activating the CCR7/Ras-ERK signaling axis

Yanfei Yin[1†], Jiajia Ma[1†], Xiaofang Lu[1], Saina Yan[1], Qianqian Jiang[1], Dazhi Wu[3], Bin Chen[1], Bo Weng[2*], MaoLiang Ran[1*]

Figure S2 The validation of primer specificity, cDNA quality, and annealing temperature.

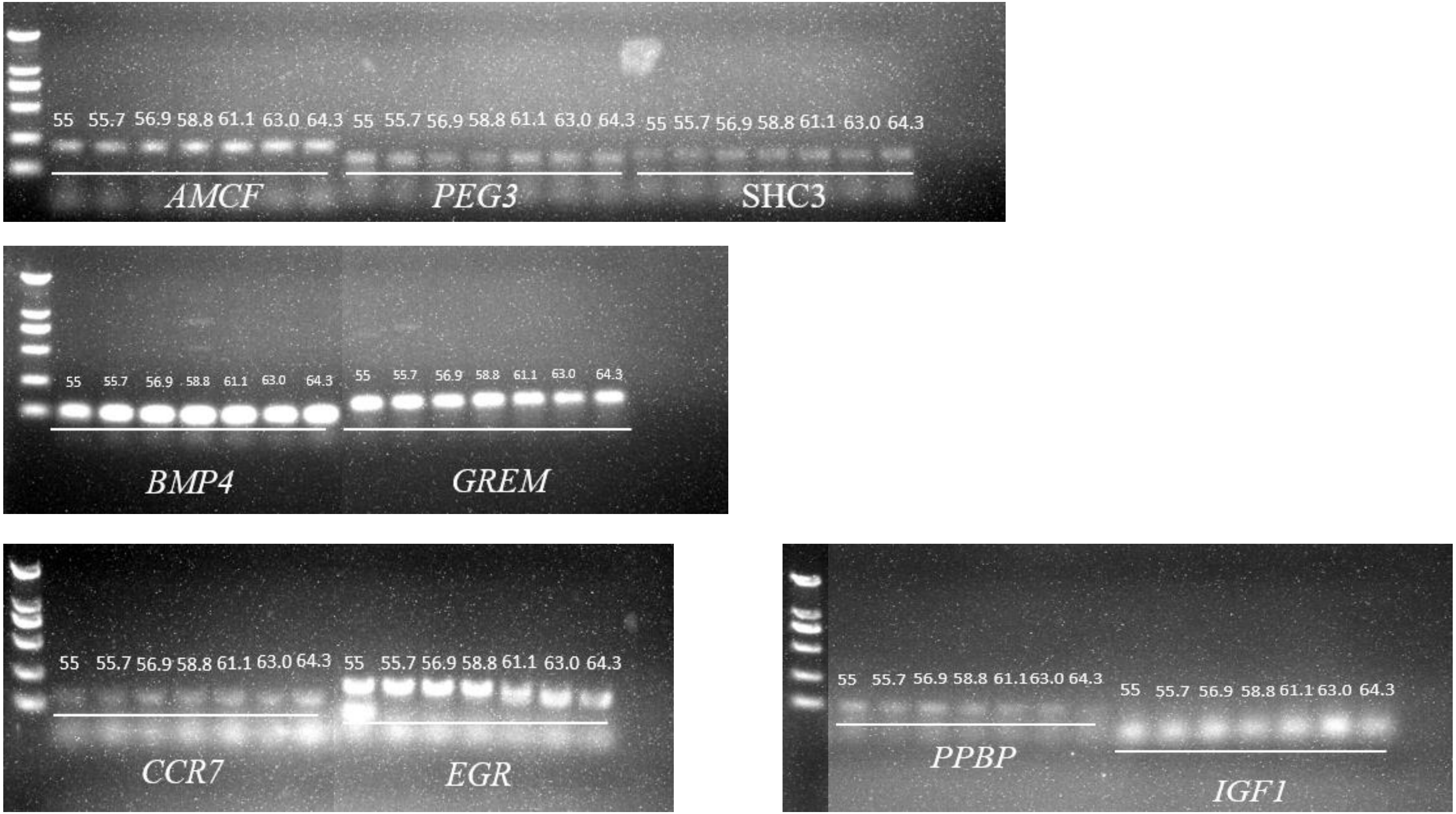
55 55.7 56.9 58.8 61.1 63.0 64.3 55 55.7 56.9 58.8 61.1 63.0 64.3 55 55.7 56.9 58.8 61.1 63.0 64.3
AMCF
PEG3
SHC3
55 55.7 56.9 58.8 61.1 63.0 64.3 55 55.7 56.9 58.8 61.1 63.0 64.3
BMP4
GREM
55 55.7 56.9 58.8 61.1 63.0 64.3 55 55.7 56.9 58.8 61.1 63.0 64.3
CCR7
EGR
55 55.7 56.9 58.8 61.1 63.0 64.3 55 55.7 56.9 58.8 61.1 63.0 64.3
PPBP
IGF1

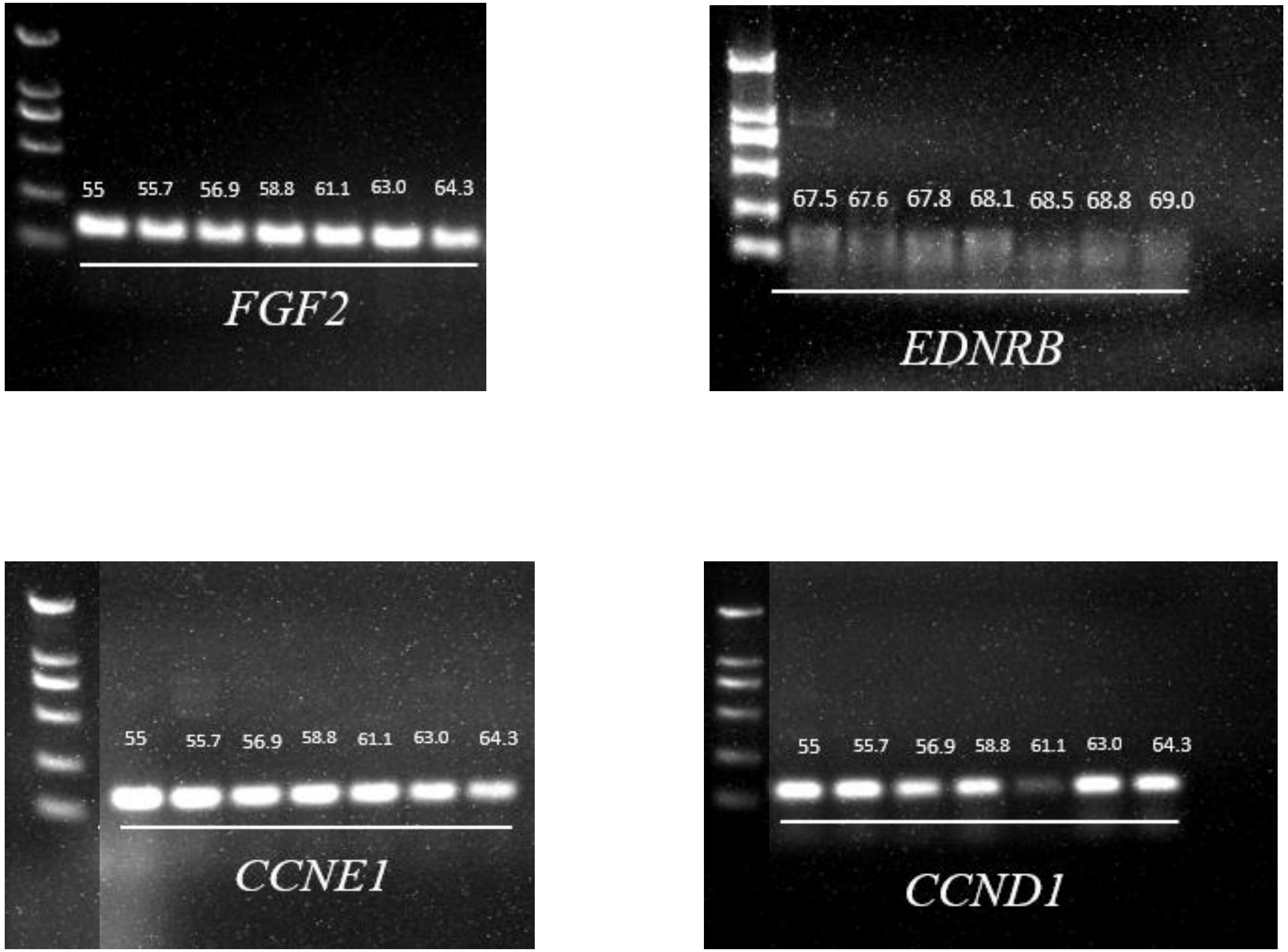
55 55.7 56.9 58.8 61.1 63.0 64.3
FGF2
67.5 67.6 67.8 68.1 68.5 68.8 69.0
EDNRB
55 55.7 56.9 58.8 61.1 63.0 64.3
CCNE1
55 55.7 56.9 58.8 61.1 63.0 64.3
CCND1

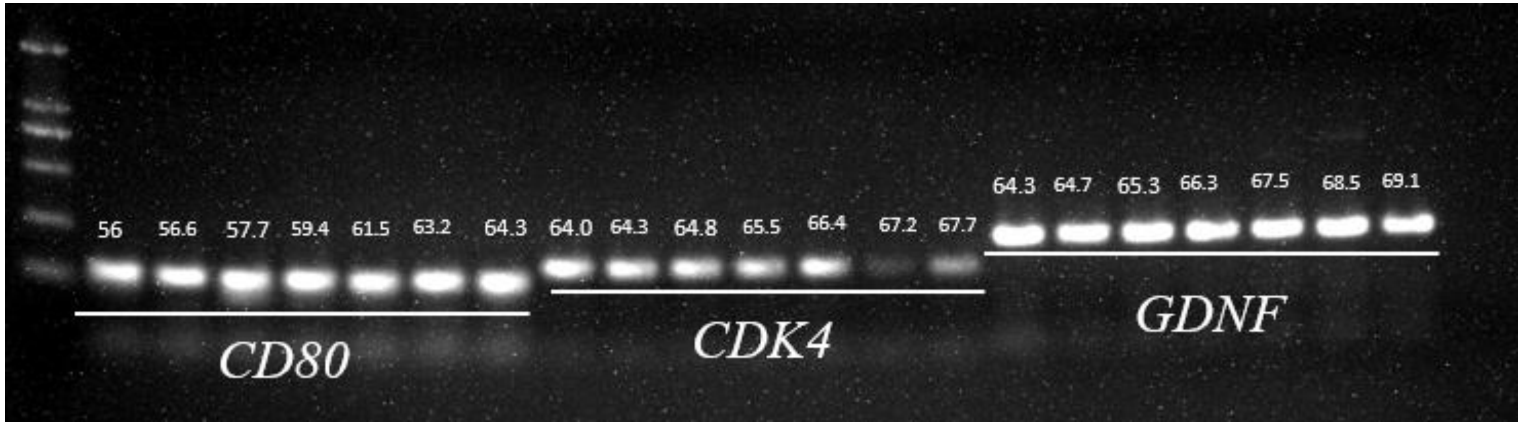
56
56.6
57.7
59.4
61.5
63.2
64.3
64.0
64.3
64.8
65.5
66.4
67.2
67.7
64.3
64.7
65.3
66.3
67.5
68.5
69.1
CD80
CDK4
GDNF

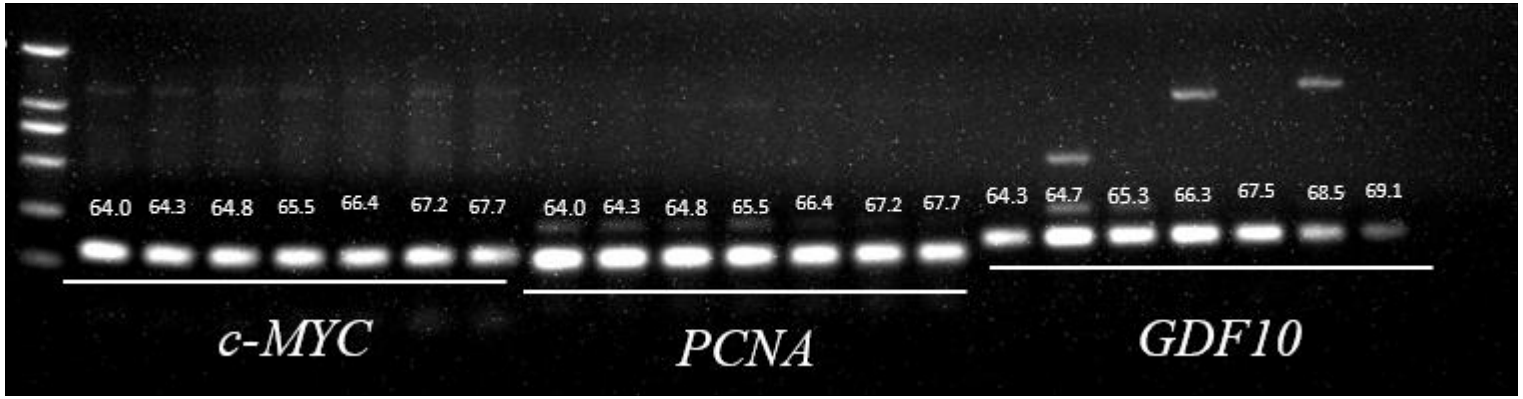
64.0
64.3
64.8
65.5
66.4
67.2
67.7
64.0
64.3
64.8
65.5
66.4
67.2
67.7
64.3
64.7
65.3
66.3
67.5
68.5
69.1
c-MYC
PCNA
GDF10
