# Supplementary material for: FSH promotes immature porcine Sertoli cell proliferation by activating the CCR7/Ras-ERK signaling axis
Source: Reproduction. 2023 Apr 26;165(6):593–603. doi: 10.1530/REP-22-0441 (PMC10235919; doi:10.1530/REP-22-0441)

FSH promotes immature porcine Sertoli cell proliferation by activating the CCR7/Ras-ERK signaling axis

Yanfei Yin[1†], Jiajia Ma[1†], Xiaofang Lu[1], Saina Yan[1], Qianqian Jiang[1], Dazhi Wu[3], Bin Chen[1], Bo Weng[2*], MaoLiang Ran[1*]

Figure S3 The melt curve of each gene in the qRT-PCR assay.

EGR1

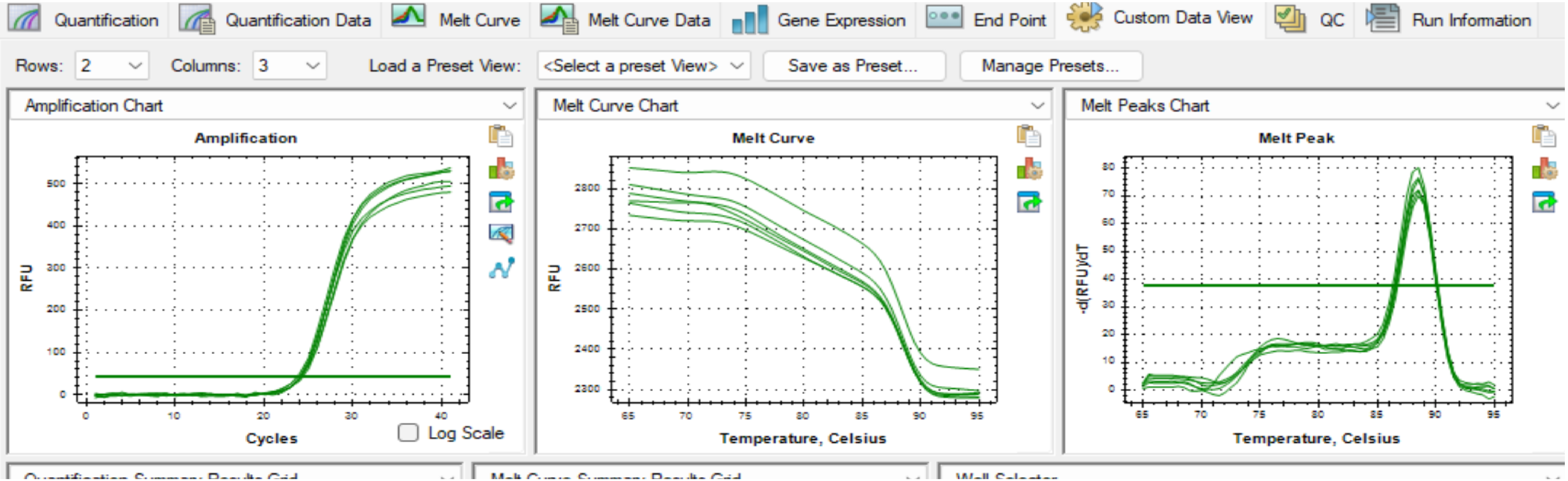
Quantification
Quantification Data
Melt Curve
Melt Curve Data
Gene Expression
End Point
Custom Data View
QC
Run Information
Rows: 2
Columns: 3
Load a Preset View:
<Select a preset View>
Save as Preset...
Manage Presets...
Amplification Chart
Amplification
RFU
Cycles
Log Scale
Melt Curve Chart
Melt Curve
RFU
Temperature, Celsius
Melt Peaks Chart
Melt Peak
-d(RFU)/dT
Temperature, Celsius

GREM1

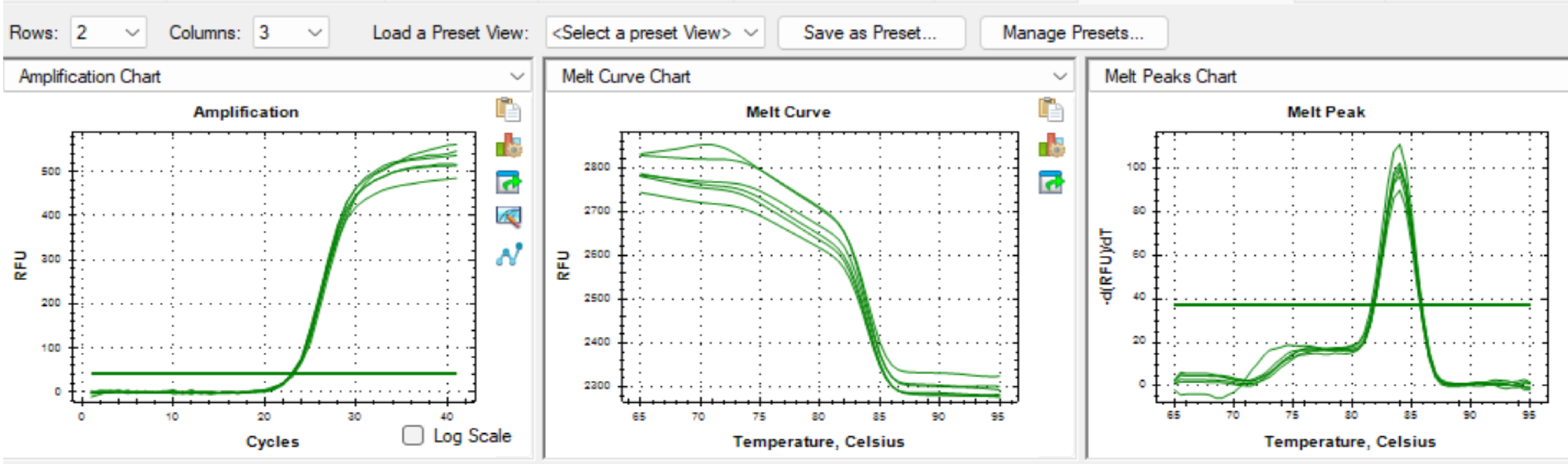

Rows: 2
Columns: 3
Load a Preset View:
<Select a preset View>
Save as Preset...
Manage Presets...
Amplification Chart
Amplification
RFU
0
100
200
300
400
500
0
10
20
30
40
Cycles
Log Scale
Melt Curve Chart
Melt Curve
RFU
2300
2400
2500
2600
2700
2800
65
70
75
80
85
90
95
Temperature, Celsius
Melt Peaks Chart
Melt Peak
-d(RFU)/dT
0
20
40
60
80
100
65
70
75
80
85
90
95
Temperature, Celsius

PEG3

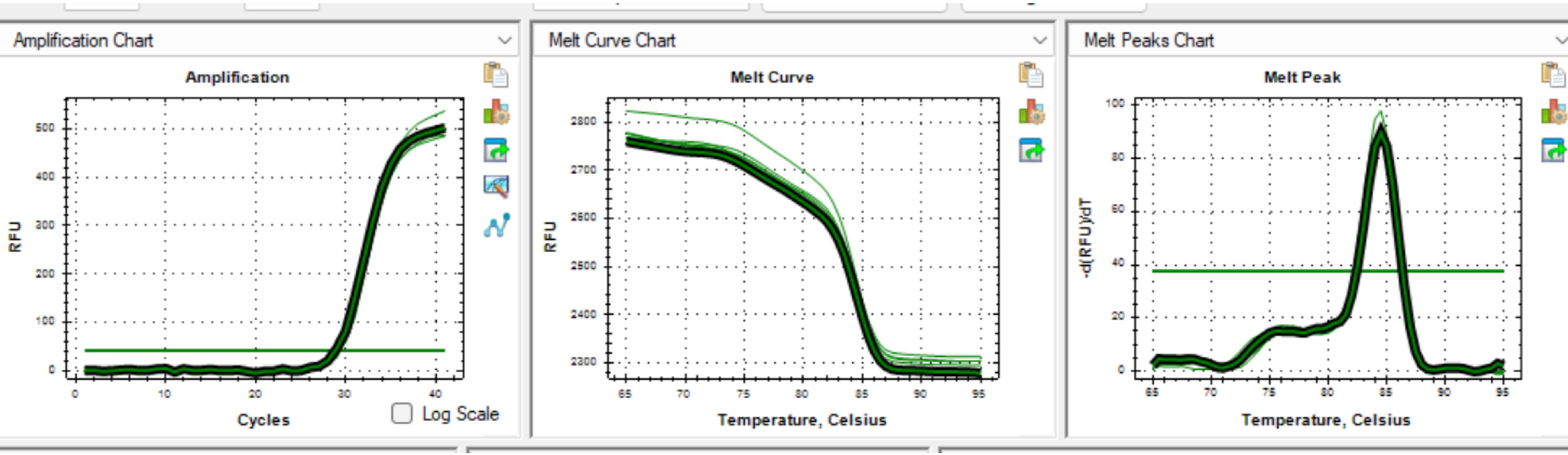

Amplification Chart
Amplification
RFU
Cycles
Log Scale
Melt Curve Chart
Melt Curve
RFU
Temperature, Celsius
Melt Peaks Chart
Melt Peak
-d(RFU)/dT
Temperature, Celsius

AMCFII

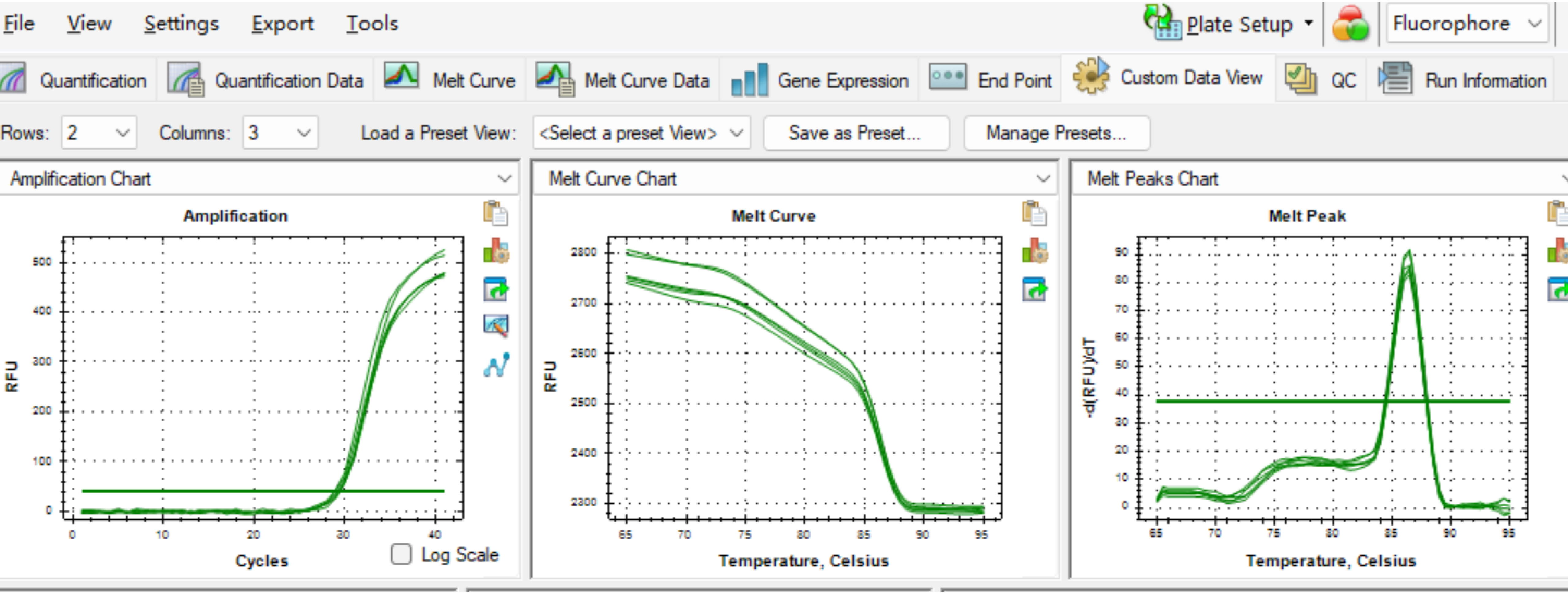

File
View
Settings
Export
Tools
Plate Setup
Fluorophore
Quantification
Quantification Data
Melt Curve
Melt Curve Data
Gene Expression
End Point
Custom Data View
QC
Run Information
Rows: 2
Columns: 3
Load a Preset View:
<Select a preset View>
Save as Preset...
Manage Presets...
Amplification Chart
Amplification
RFU
Cycles
Log Scale
Melt Curve Chart
Melt Curve
RFU
Temperature, Celsius
Melt Peaks Chart
Melt Peak
-d(RFU)/dT
Temperature, Celsius

CCR7

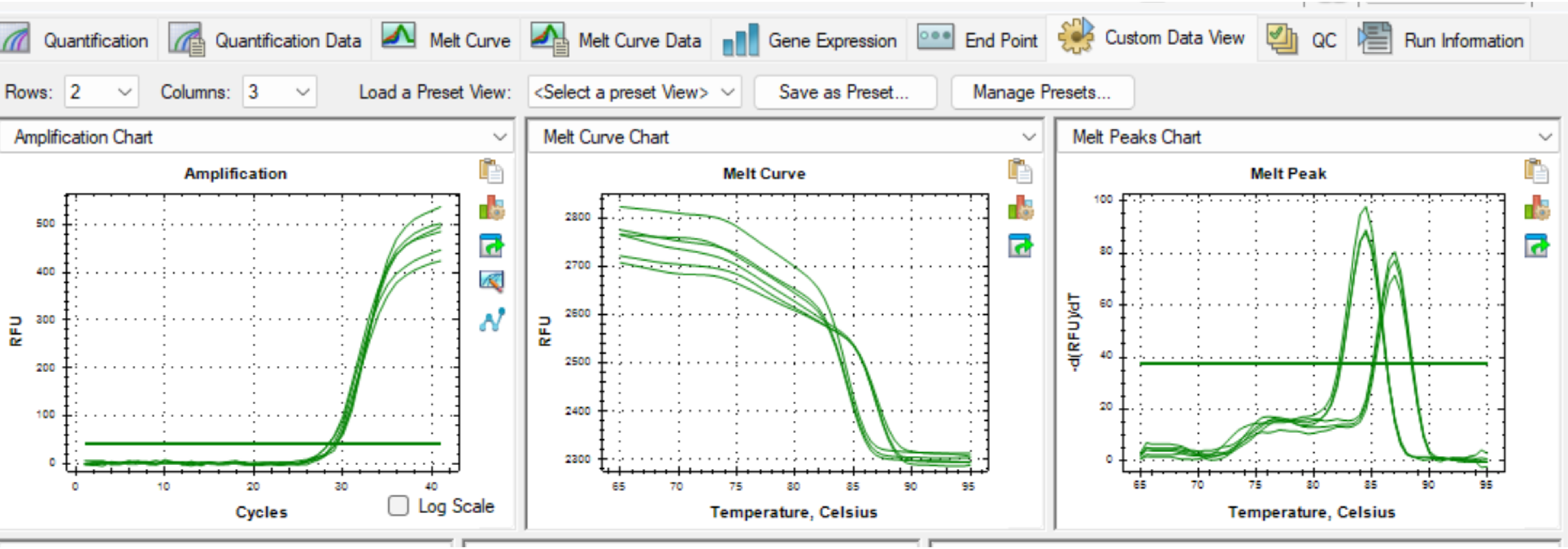
Quantification
Quantification Data
Melt Curve
Melt Curve Data
Gene Expression
End Point
Custom Data View
QC
Run Information
Rows: 2
Columns: 3
Load a Preset View:
<Select a preset View>
Save as Preset...
Manage Presets...
Amplification Chart
Amplification
RFU
Cycles
Log Scale
Melt Curve Chart
Melt Curve
RFU
Temperature, Celsius
Melt Peaks Chart
Melt Peak
-d(RFU)/dT
Temperature, Celsius

PPBP

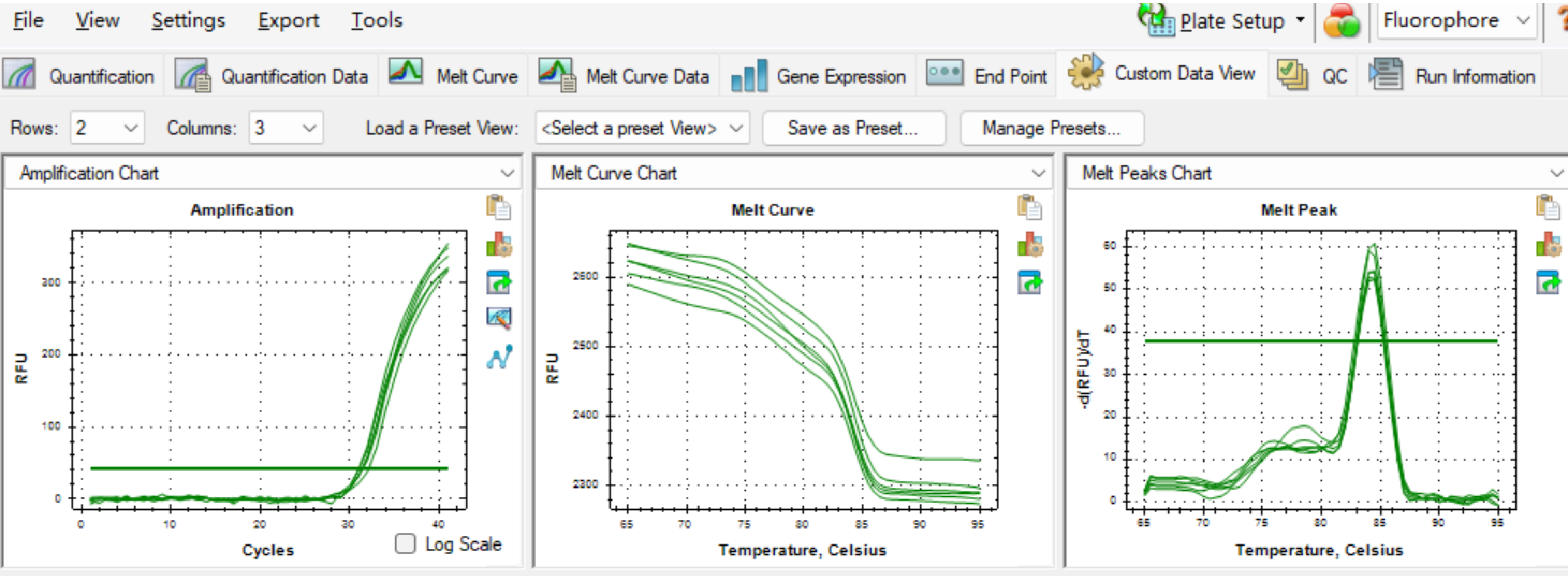
File
View
Settings
Export
Tools
Plate Setup
Fluorophore
Quantification
Quantification Data
Melt Curve
Melt Curve Data
Gene Expression
End Point
Custom Data View
QC
Run Information
Rows: 2
Columns: 3
Load a Preset View:
<Select a preset View>
Save as Preset...
Manage Presets...
Amplification Chart
Amplification
RFU
0
100
200
300
0
10
20
30
40
Cycles
Log Scale
Melt Curve Chart
Melt Curve
RFU
2300
2400
2500
2600
65
70
75
80
85
90
95
Temperature, Celsius
Melt Peaks Chart
Melt Peak
-d(RFU)/dT
0
10
20
30
40
50
60
65
70
75
80
85
90
95
Temperature, Celsius

SHC3

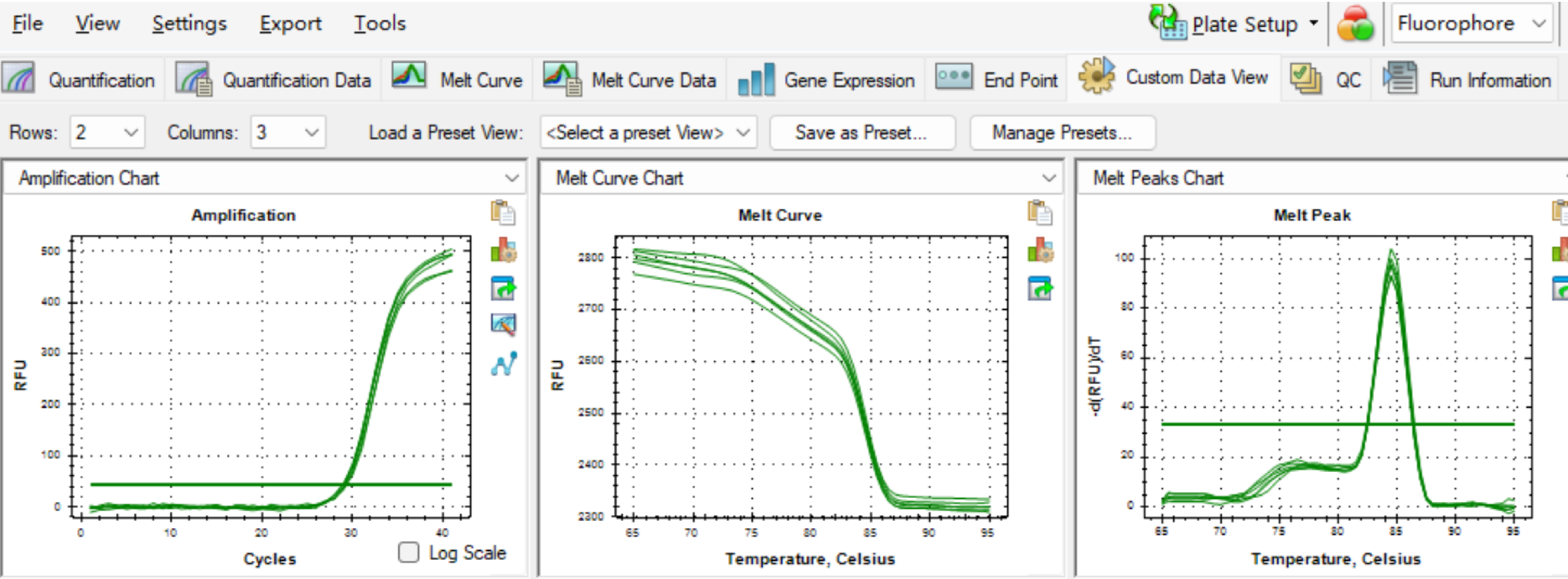
File View Settings Export Tools
Plate Setup
Fluorophore
Quantification
Quantification Data
Melt Curve
Melt Curve Data
Gene Expression
End Point
Custom Data View
QC
Run Information
Rows: 2
Columns: 3
Load a Preset View:
<Select a preset View>
Save as Preset...
Manage Presets...
Amplification Chart
Amplification
RFU
Cycles
Log Scale
Melt Curve Chart
Melt Curve
RFU
Temperature, Celsius
Melt Peaks Chart
Melt Peak
-d(RFU)/dT
Temperature, Celsius

EDNRB

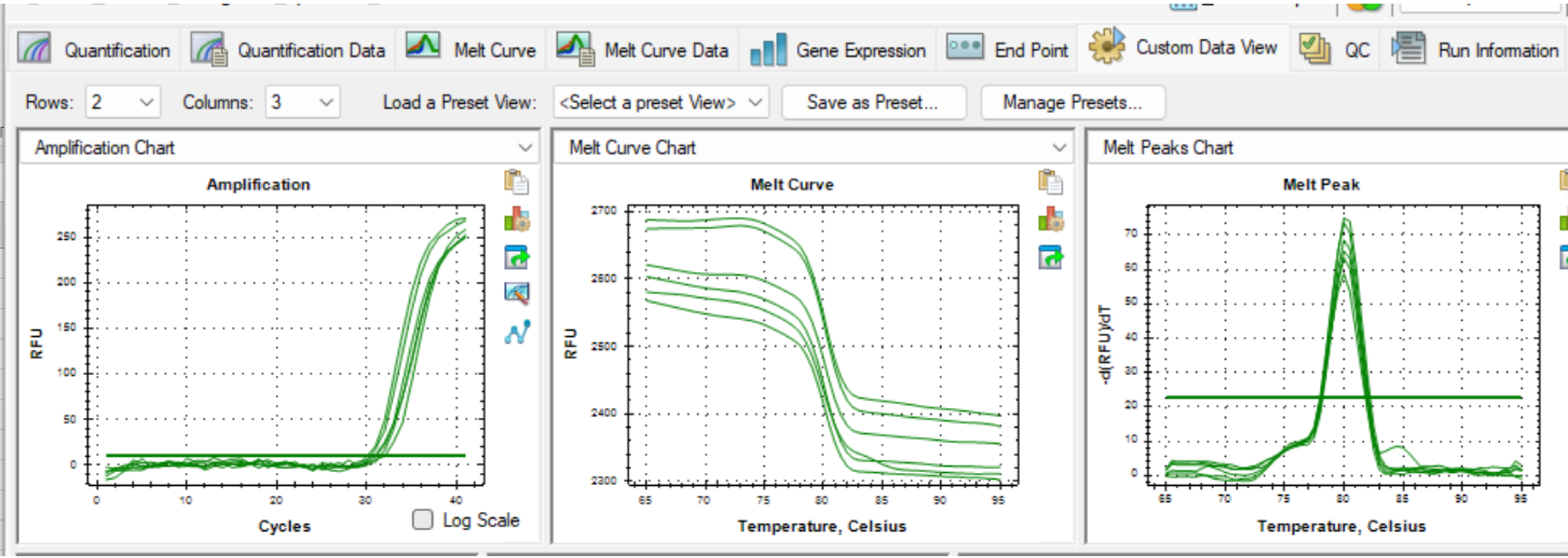

Quantification
Quantification Data
Melt Curve
Melt Curve Data
Gene Expression
End Point
Custom Data View
QC
Run Information
Rows: 2
Columns: 3
Load a Preset View:
<Select a preset View>
Save as Preset...
Manage Presets...
Amplification Chart
Amplification
RFU
Cycles
Log Scale
Melt Curve Chart
Melt Curve
RFU
Temperature, Celsius
Melt Peaks Chart
Melt Peak
-d(RFU)/dT
Temperature, Celsius

GDF10

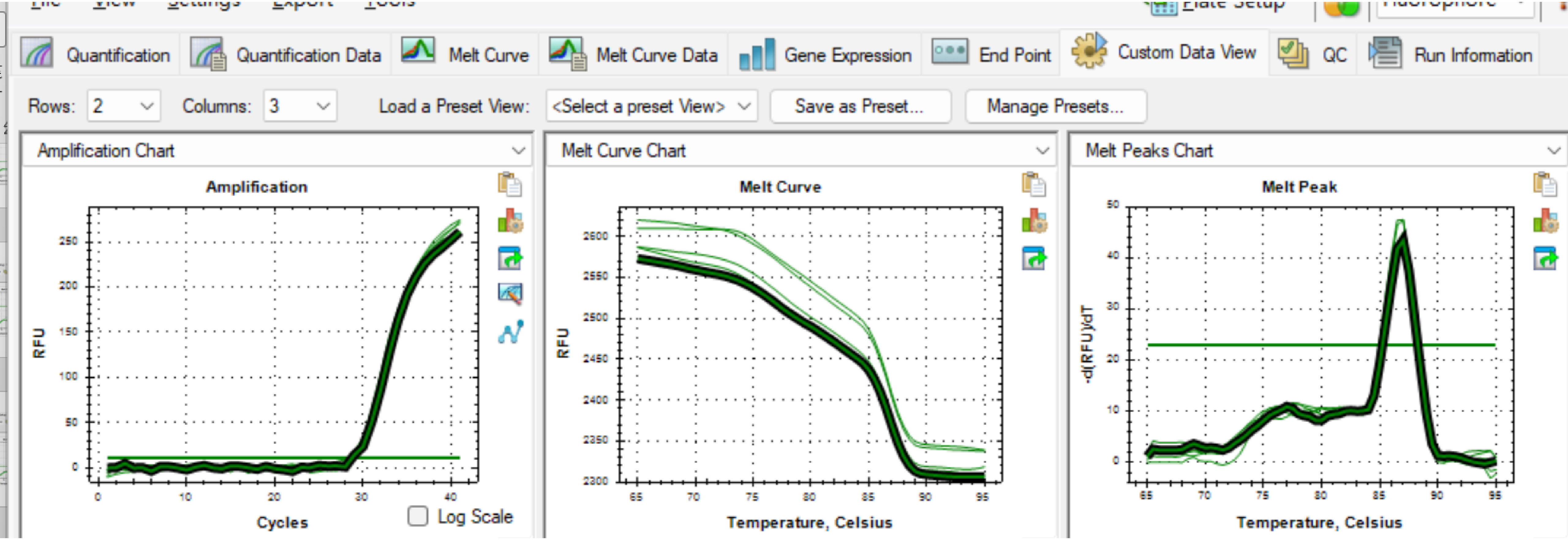
Quantification
Quantification Data
Melt Curve
Melt Curve Data
Gene Expression
End Point
Custom Data View
QC
Run Information
Rows: 2
Columns: 3
Load a Preset View:
<Select a preset View>
Save as Preset...
Manage Presets...
Amplification Chart
Amplification
RFU
Cycles
Log Scale
Melt Curve Chart
Melt Curve
Temperature, Celsius
Melt Peaks Chart
Melt Peak
-d(RFU)/dT

CD80

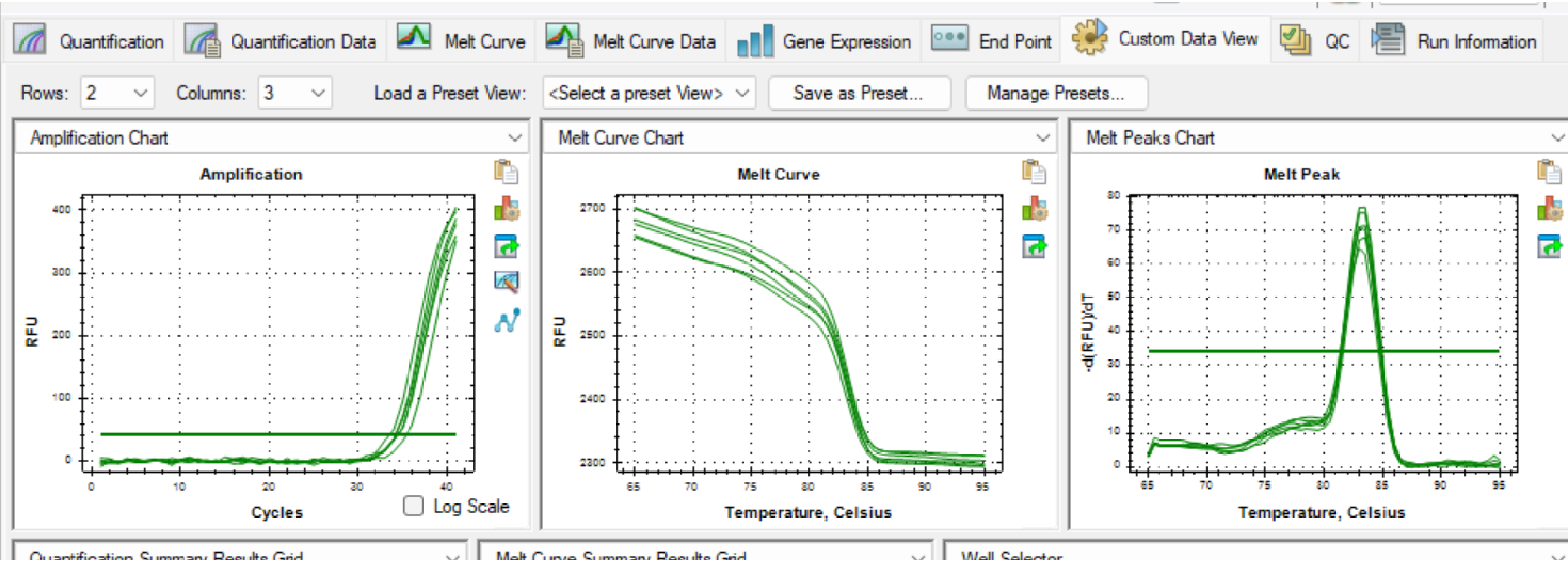
Quantification
Quantification Data
Melt Curve
Melt Curve Data
Gene Expression
End Point
Custom Data View
QC
Run Information
Rows: 2
Columns: 3
Load a Preset View:
<Select a preset View>
Save as Preset...
Manage Presets...
Amplification Chart
Amplification
RFU
Cycles
Log Scale
Melt Curve Chart
Melt Curve
RFU
Temperature, Celsius
Melt Peaks Chart
Melt Peak
-d(RFU)/dT
Temperature, Celsius

CCR7 siRNA—*CCR7*

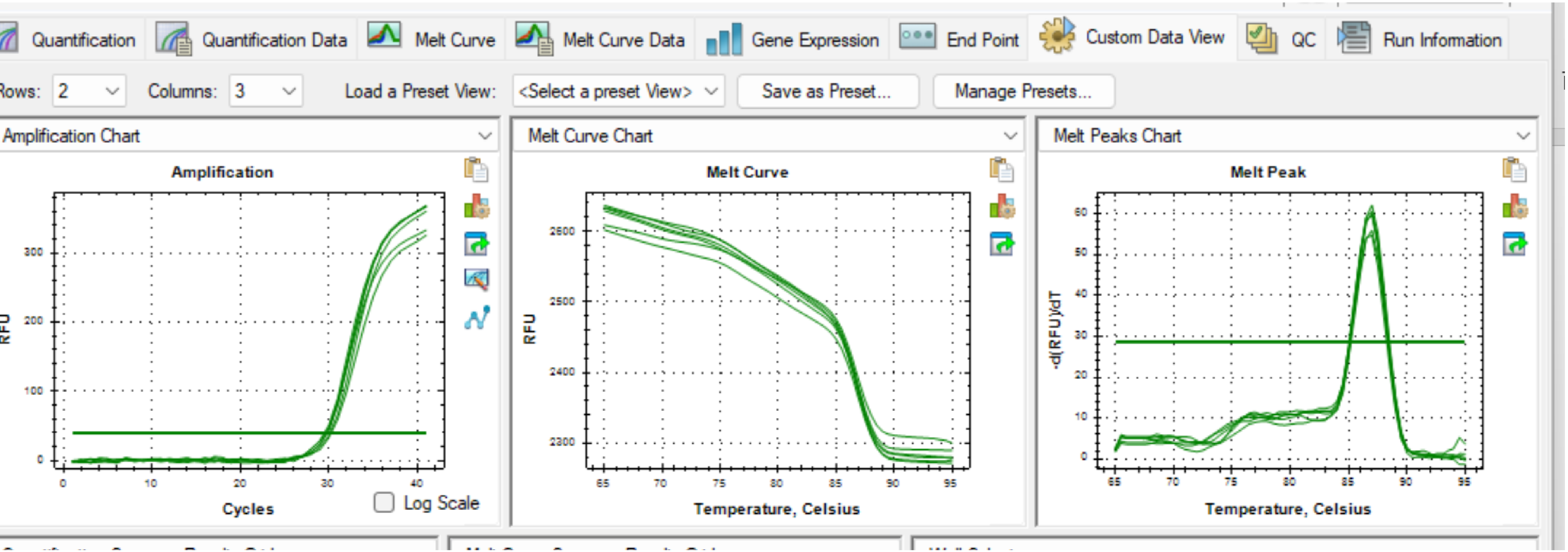

CCR7 siRNA—*BMP4*

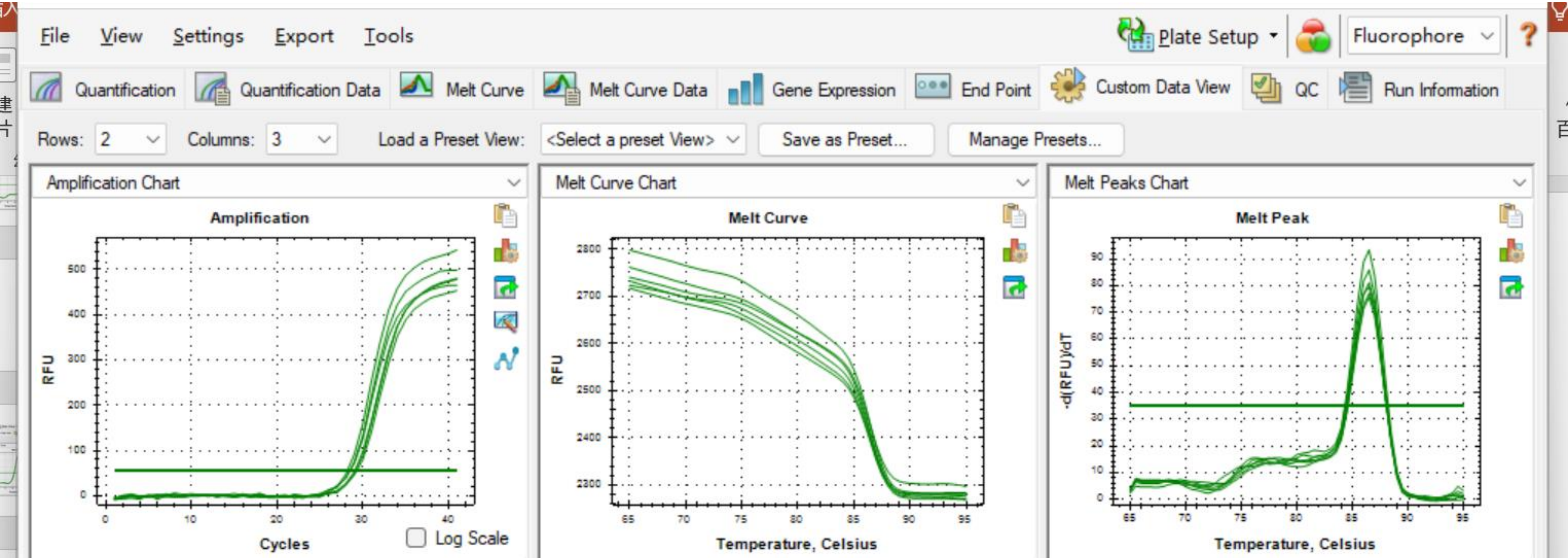

CCR7 siRNA—*IGF1*

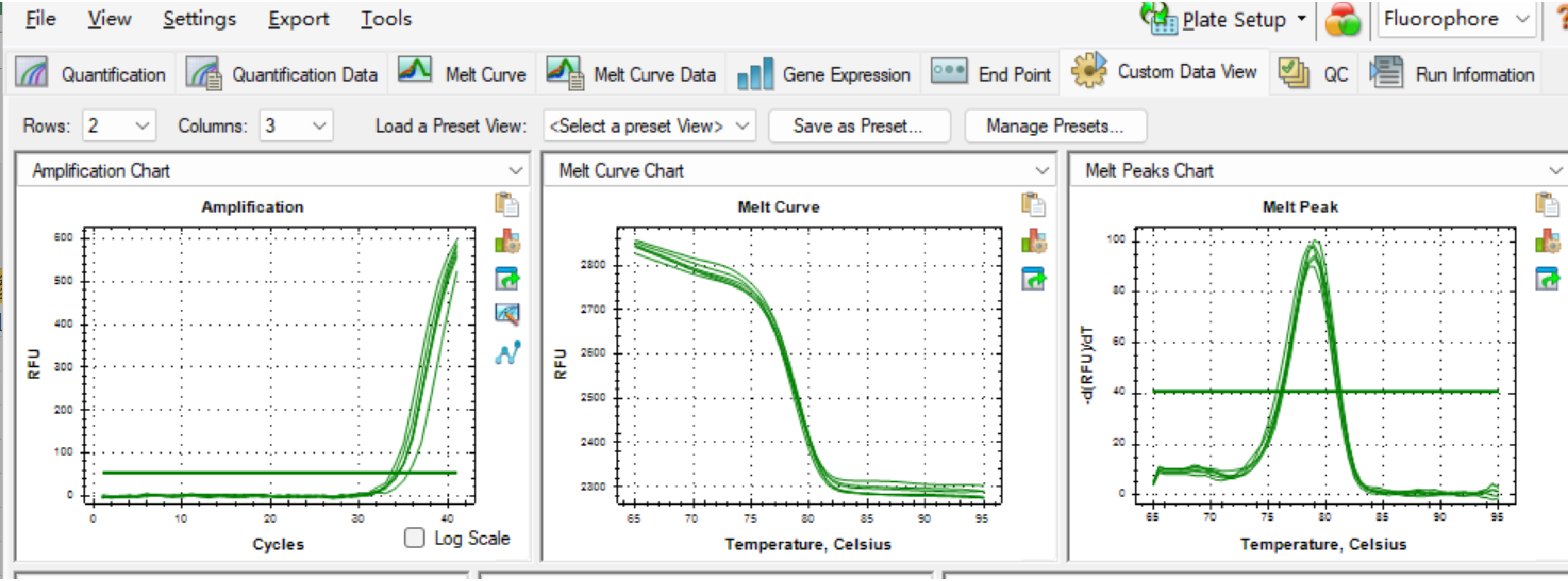

CCR7 siRNA—*FGF2*

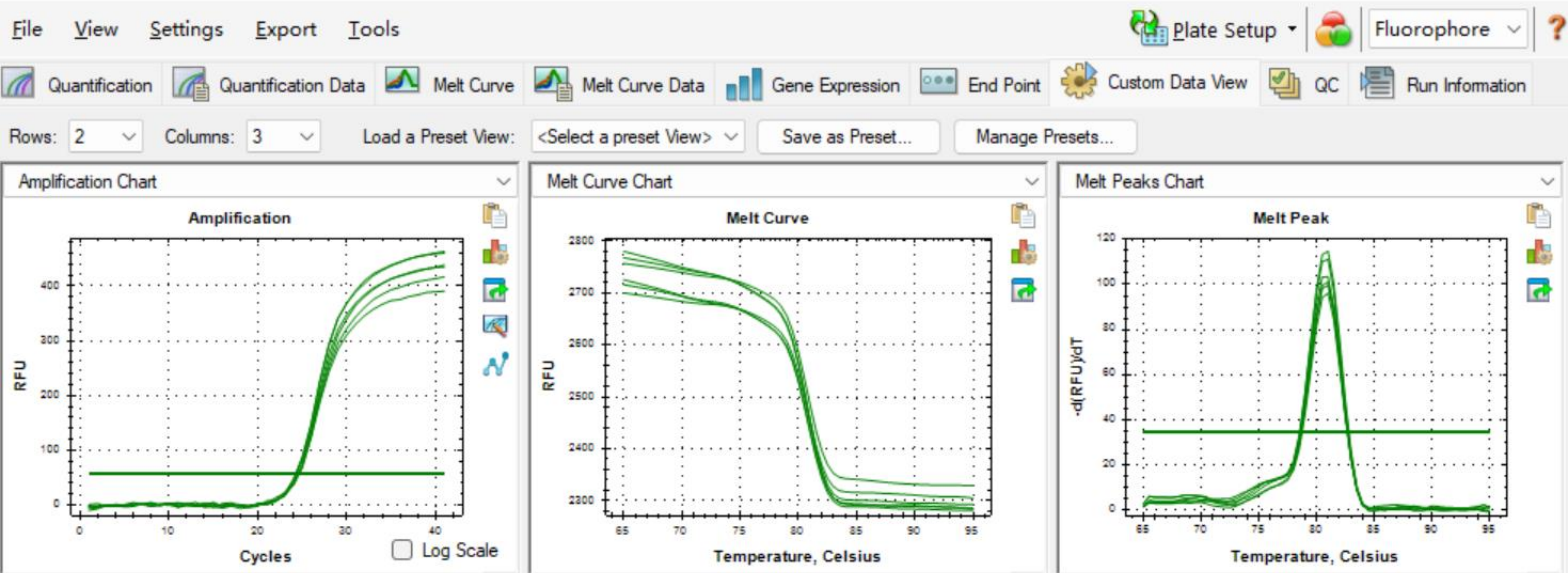

CCR7 siRNA—*GDNF*

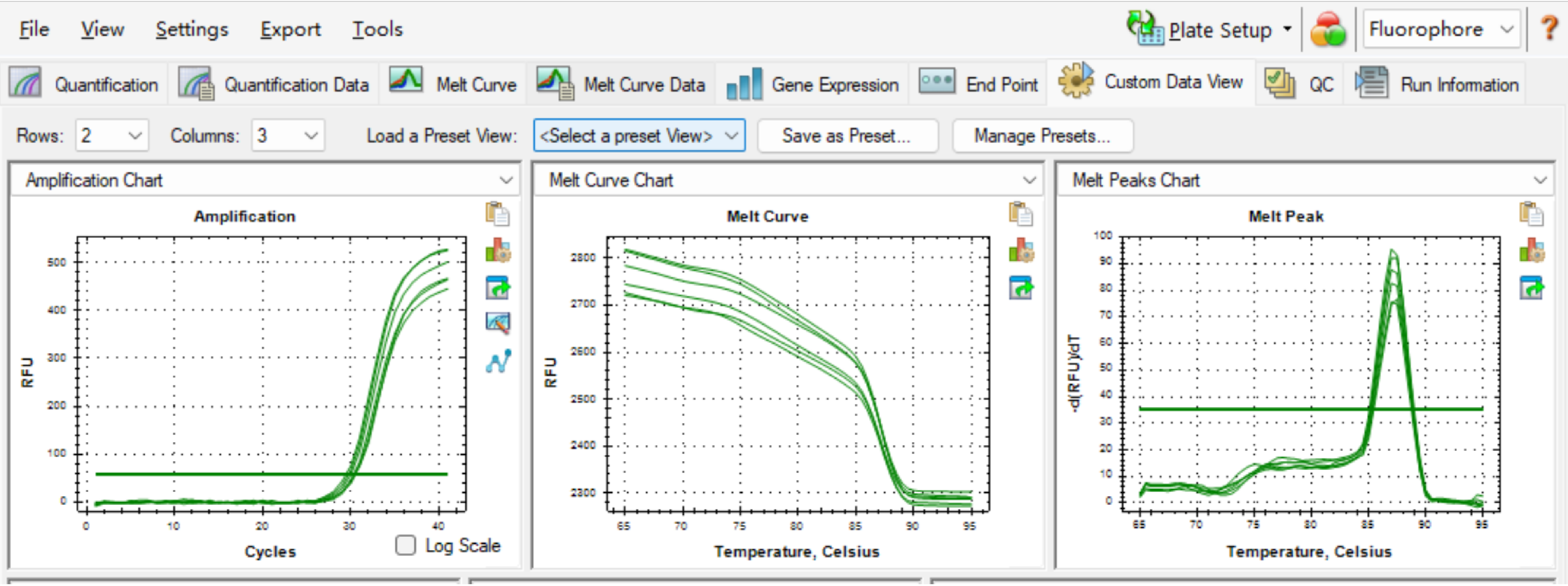

CCR7 siRNA—*PCNA*

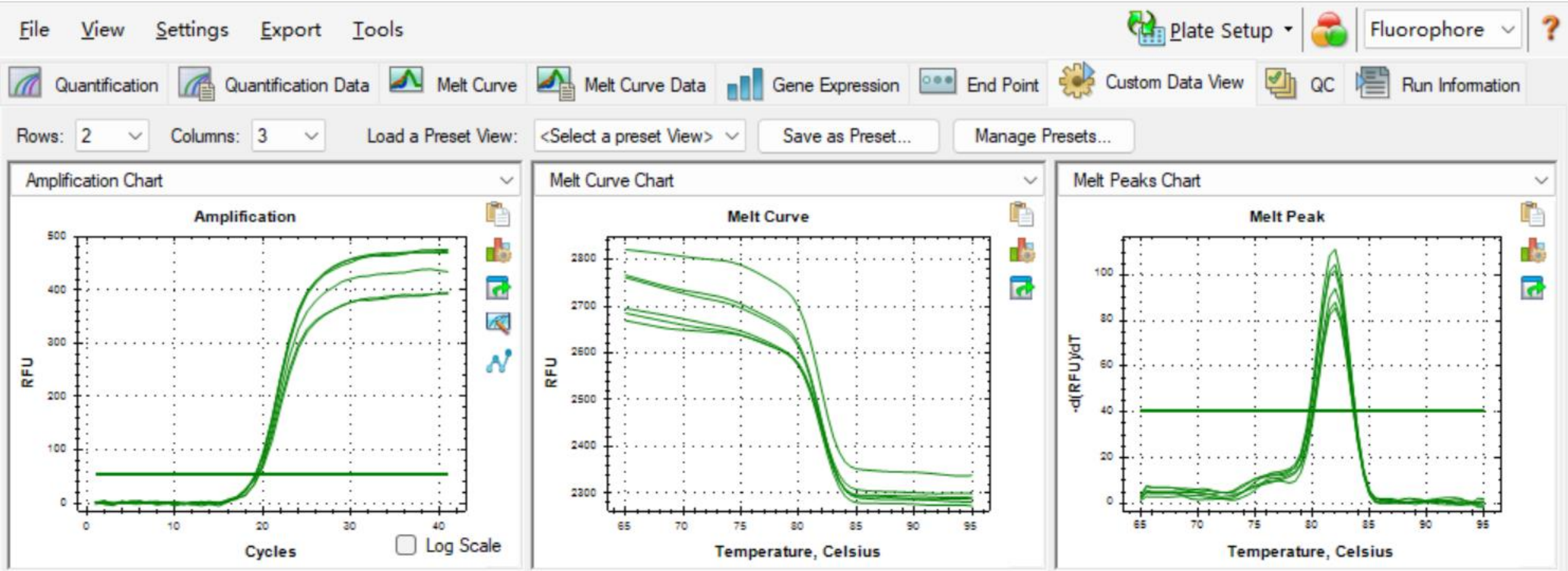

CCR7 siRNA—*MYC*

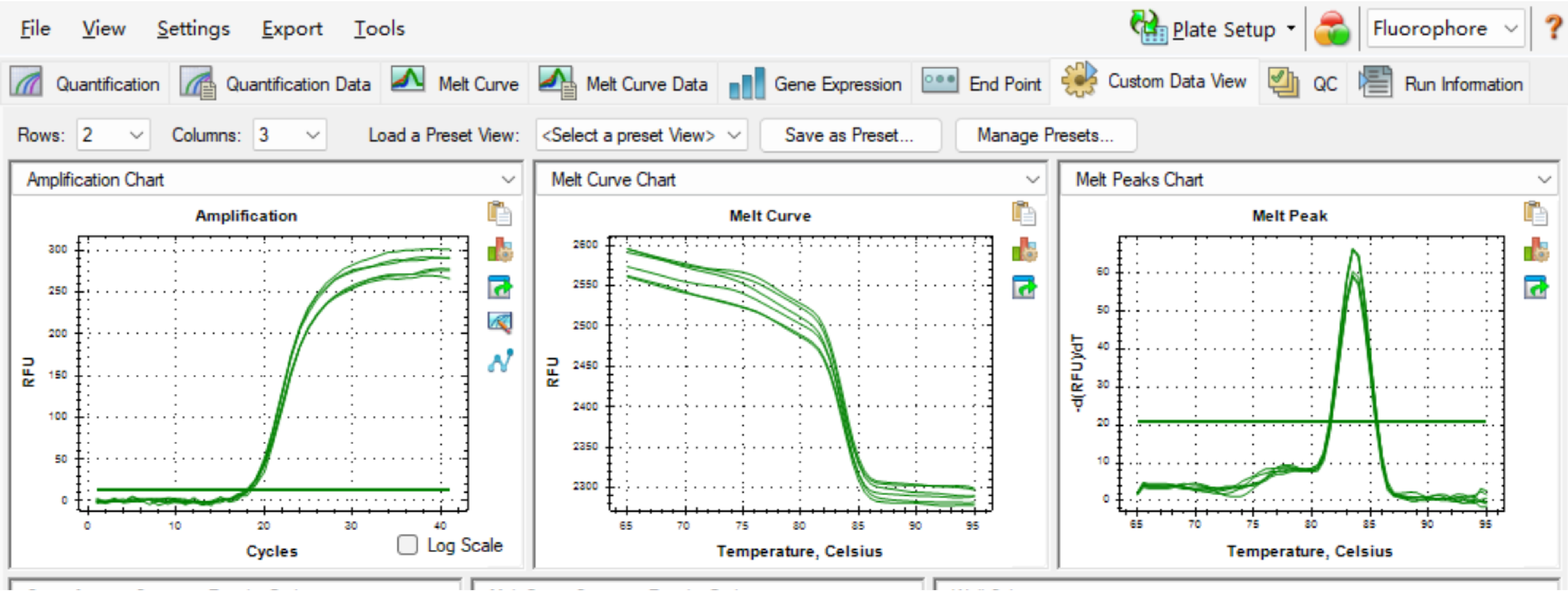

CCR7 siRNA—*CCNE1*

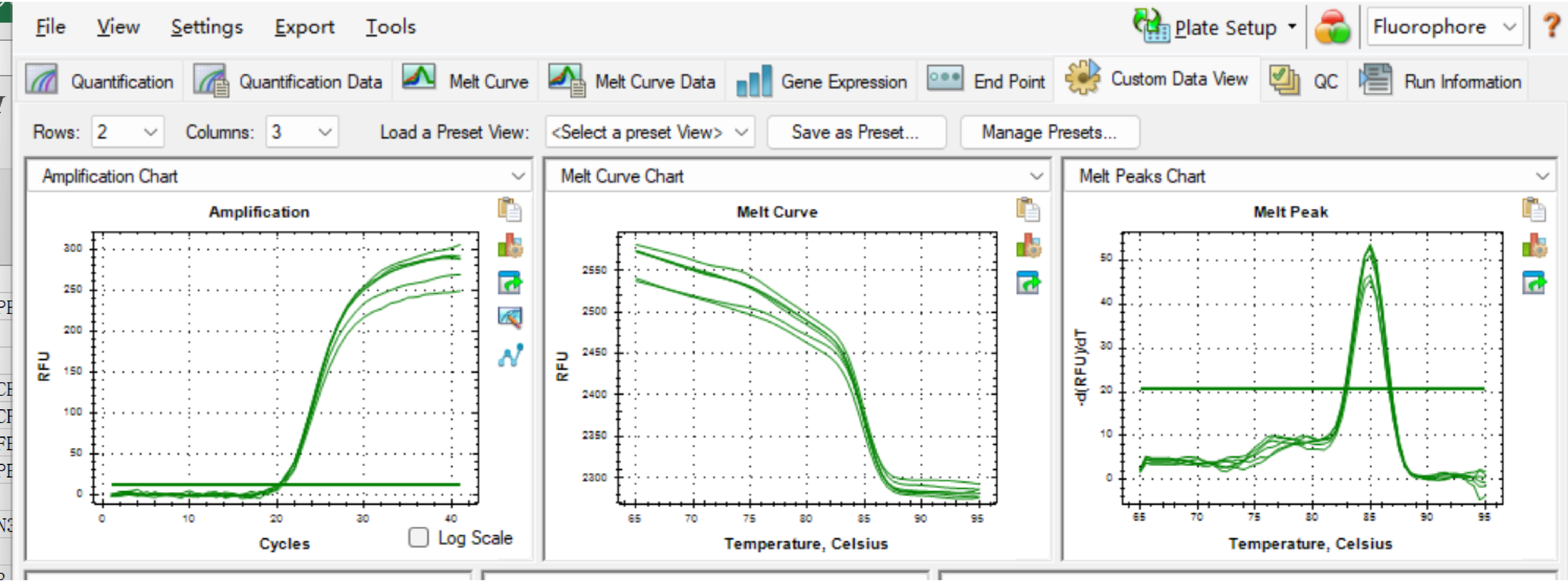

CCR7 siRNA—*CCND1*

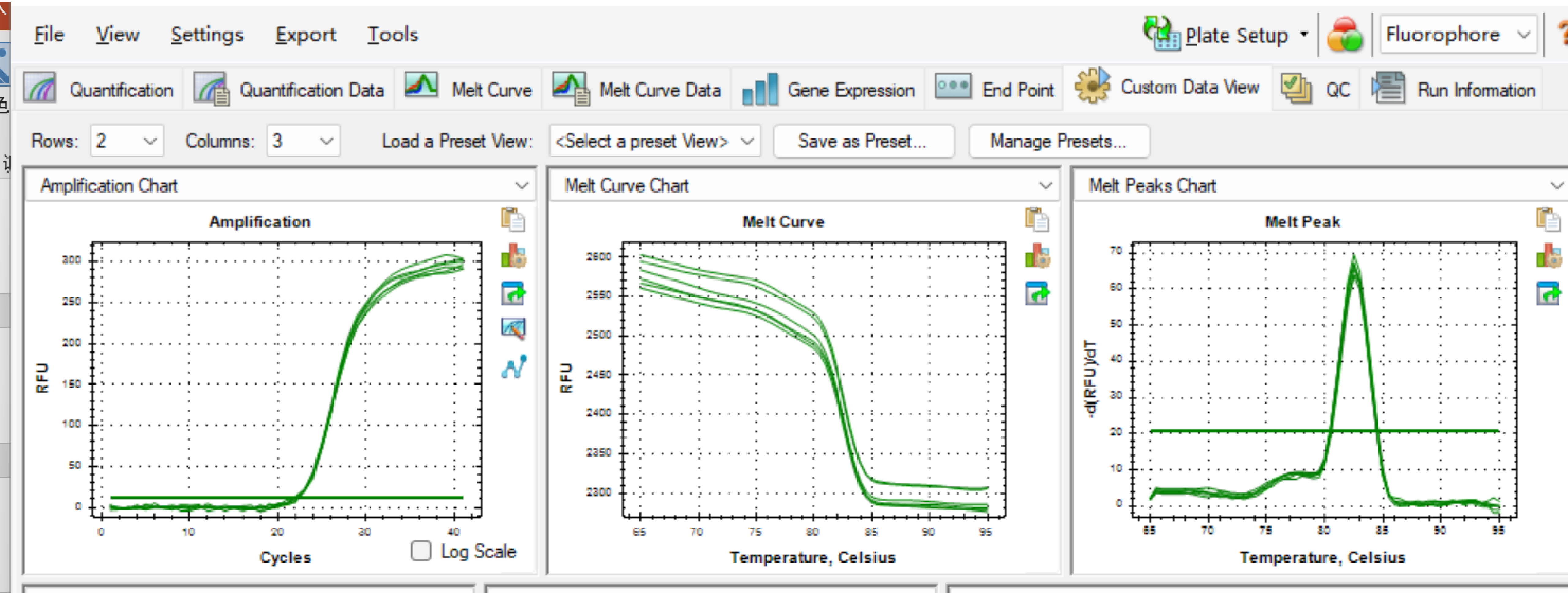

CCR7 siRNA—*CDK4*

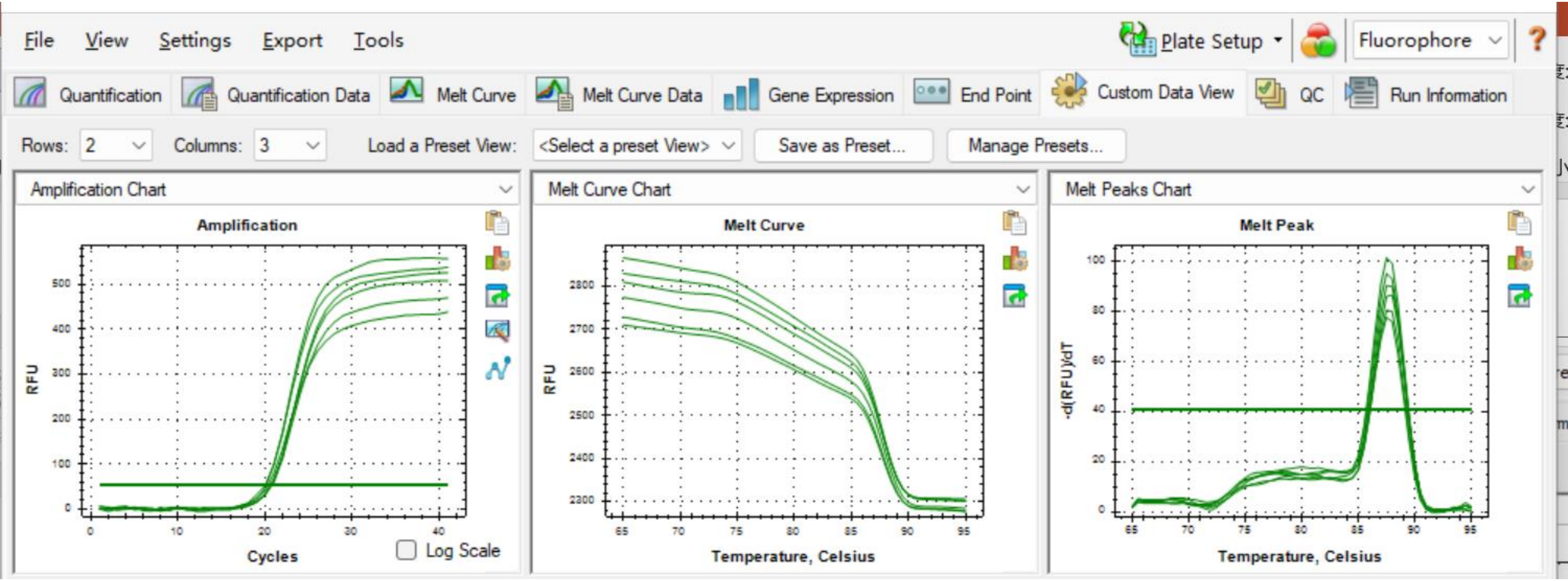

Ras inhibitor—*BMP4*

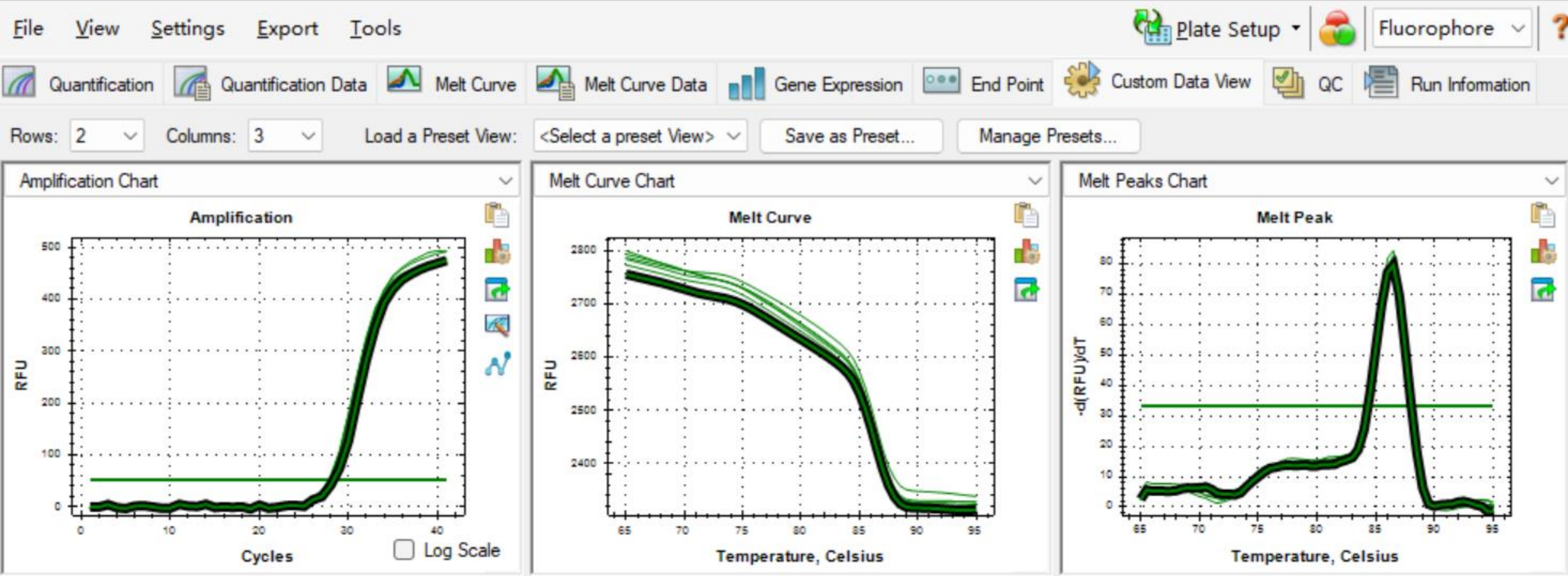

Ras inhibitor—*IGF1*

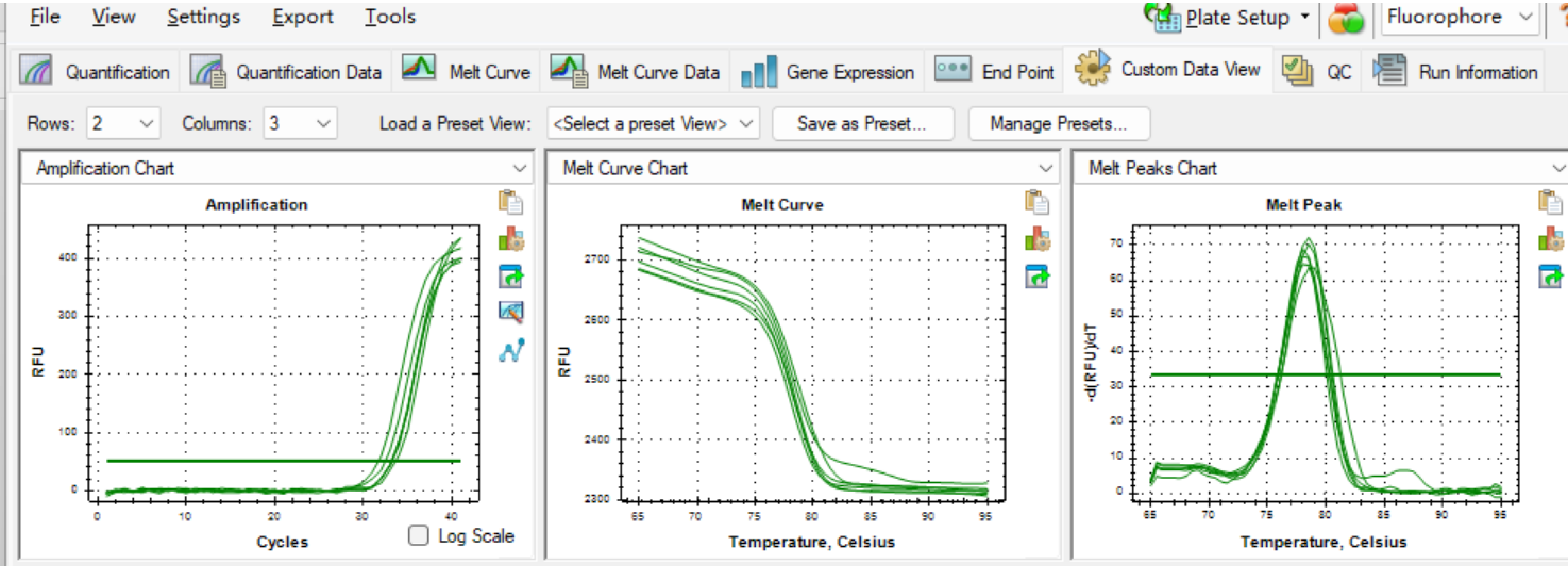

Ras inhibitor—*FGF2*

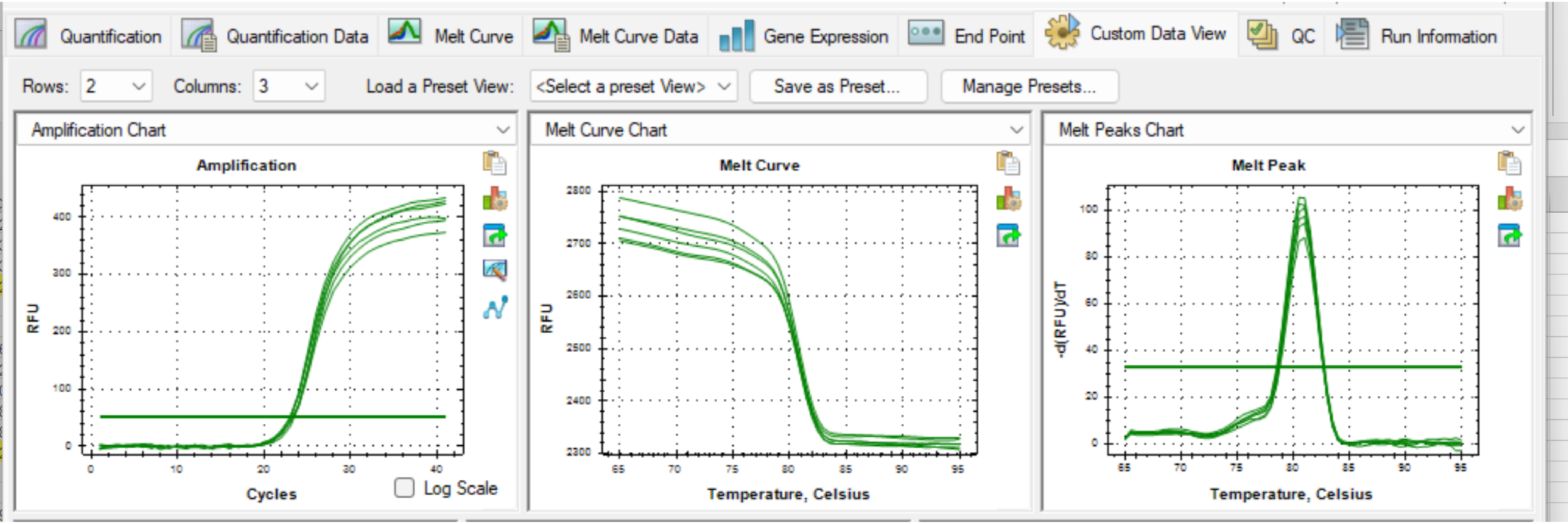

Ras inhibitor —*GDNF*

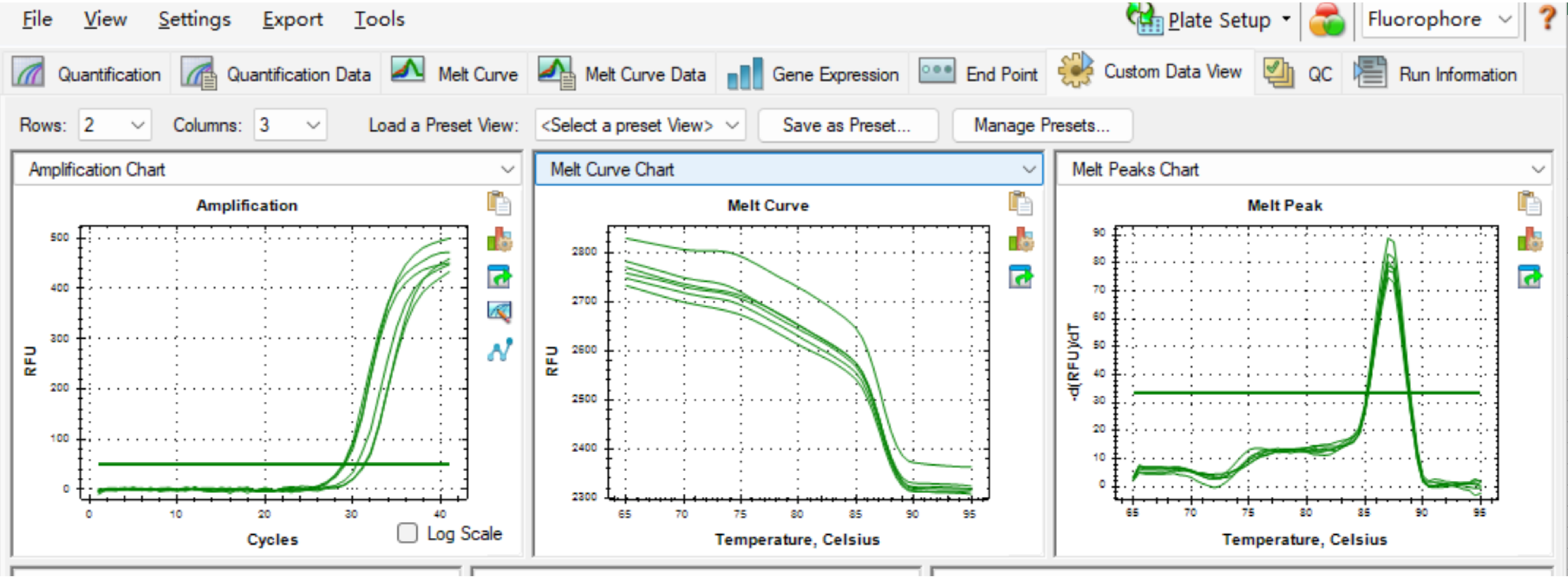

Ras inhibitor—*PCNA*

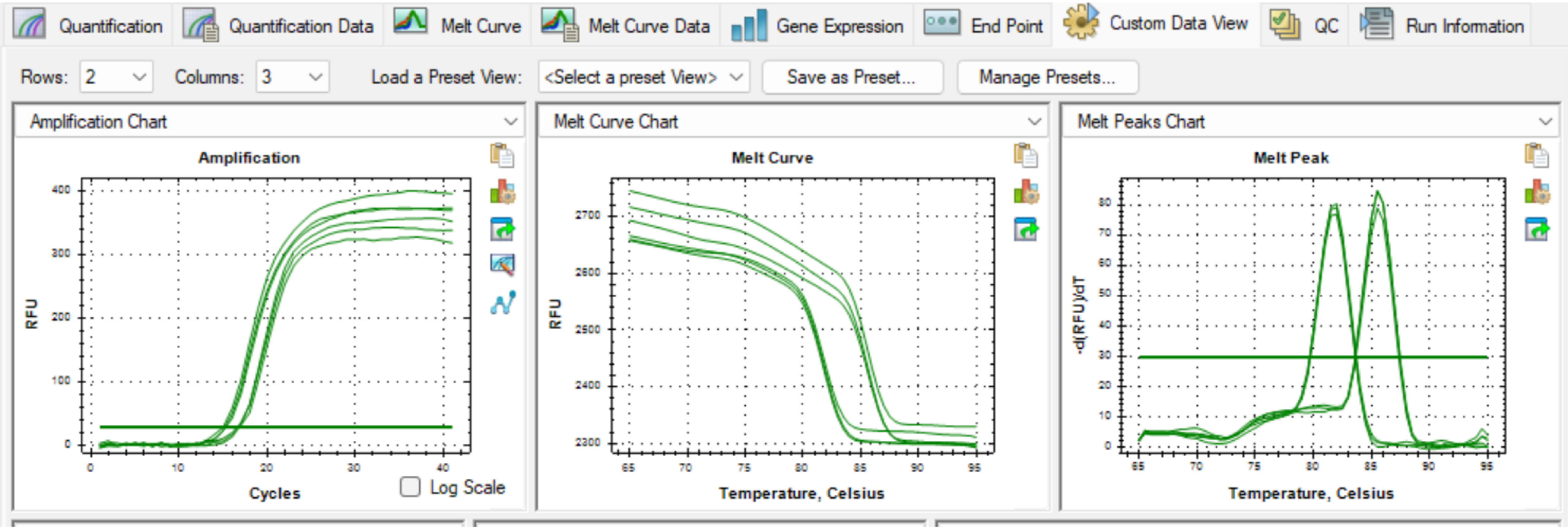

Ras inhibitor —*MYC*

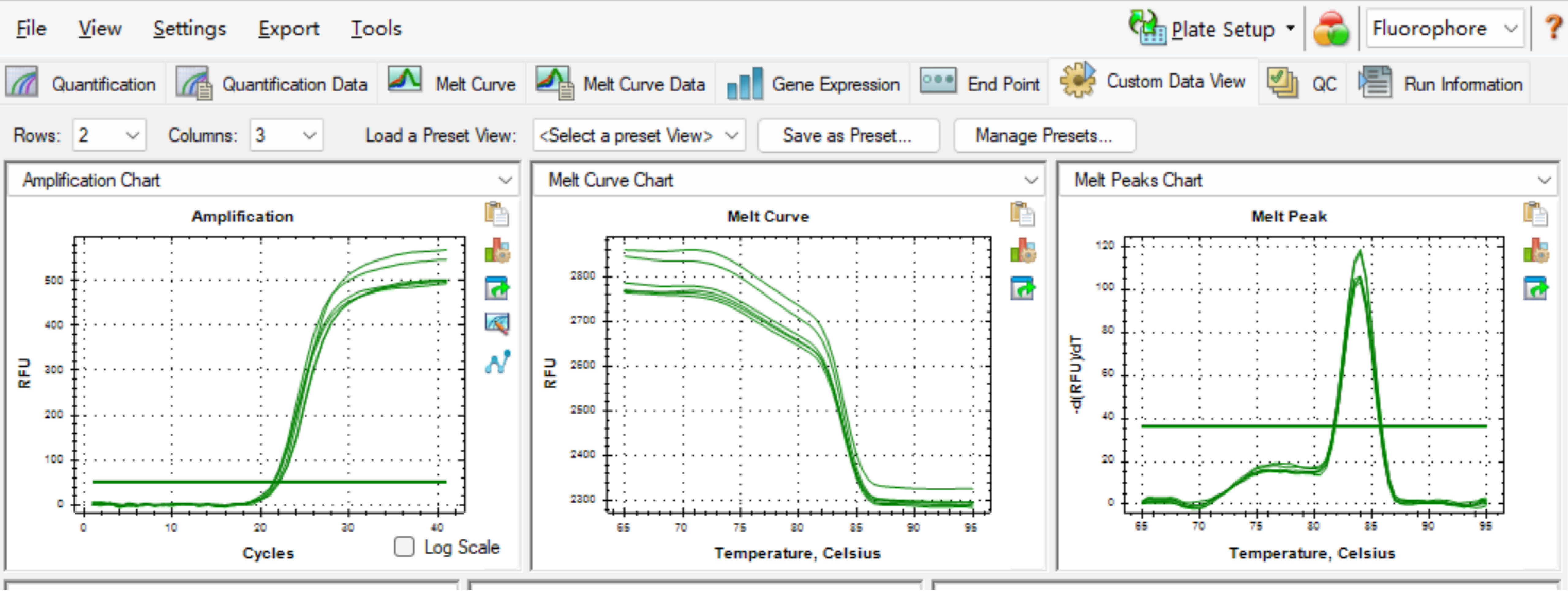

Ras inhibitor—*CCND1*

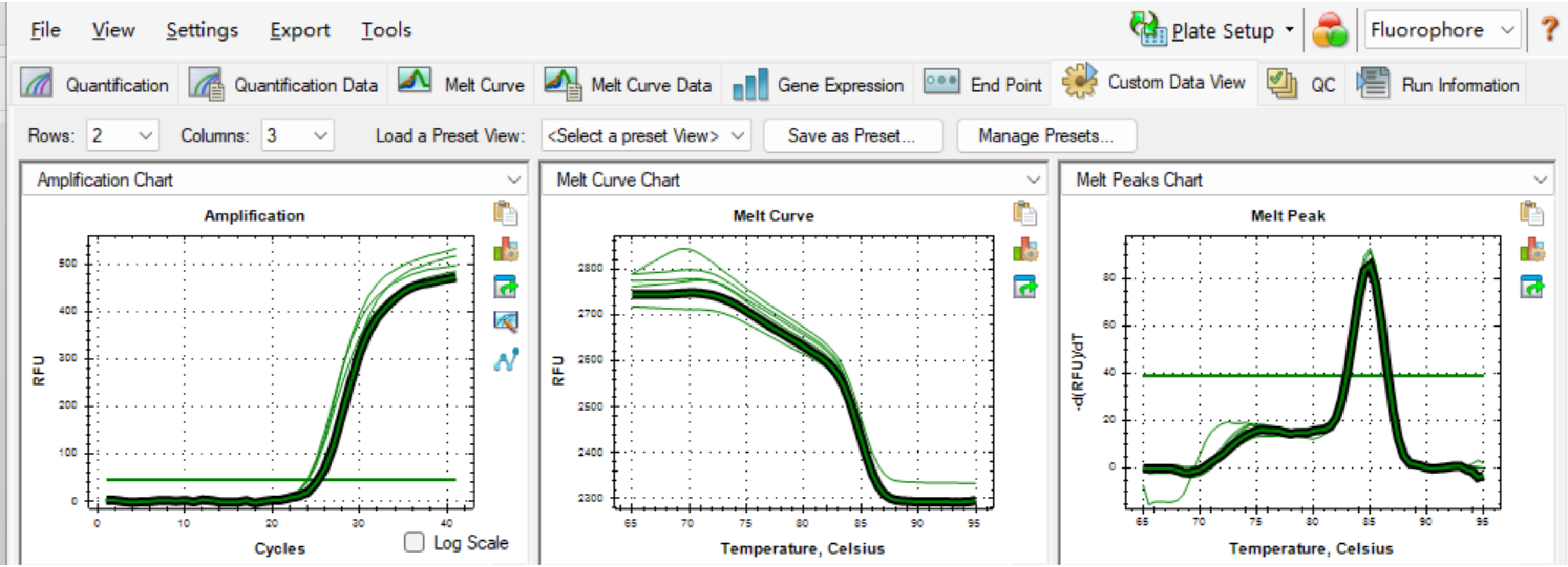

Ras inhibitor—*CCNE1*

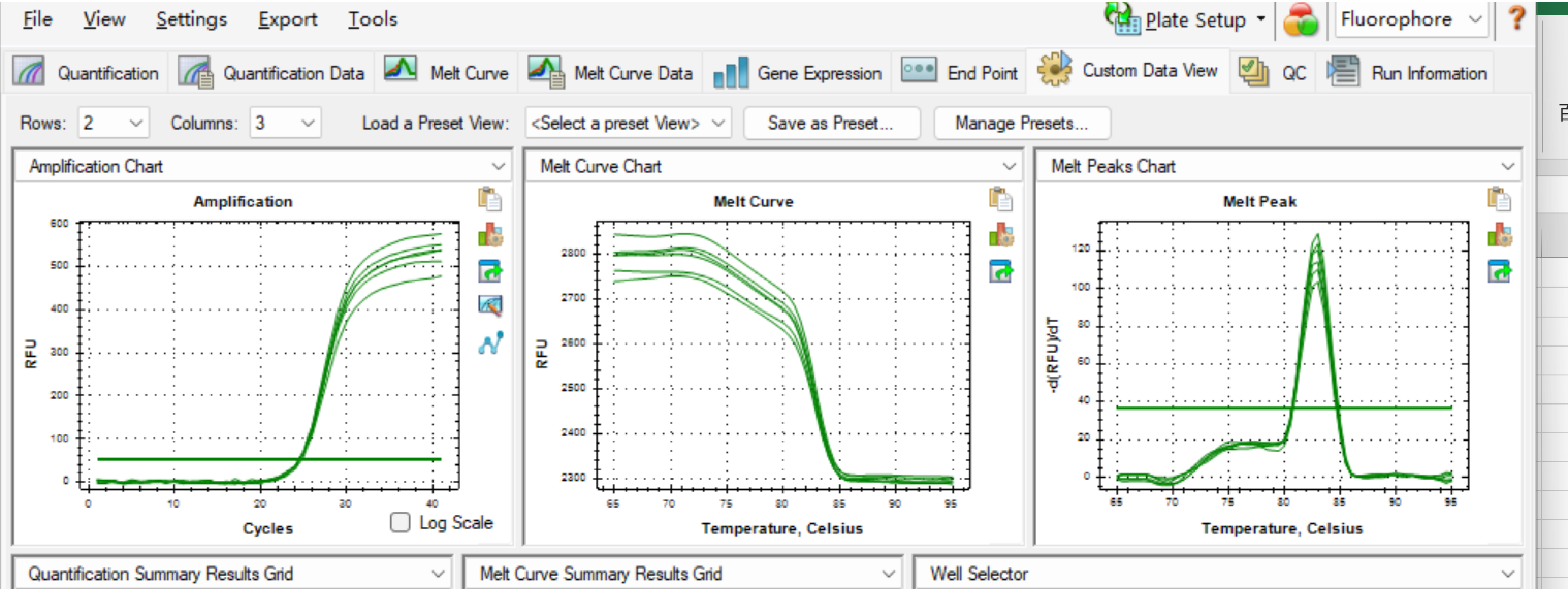

Ras inhibitor—*CDK4*

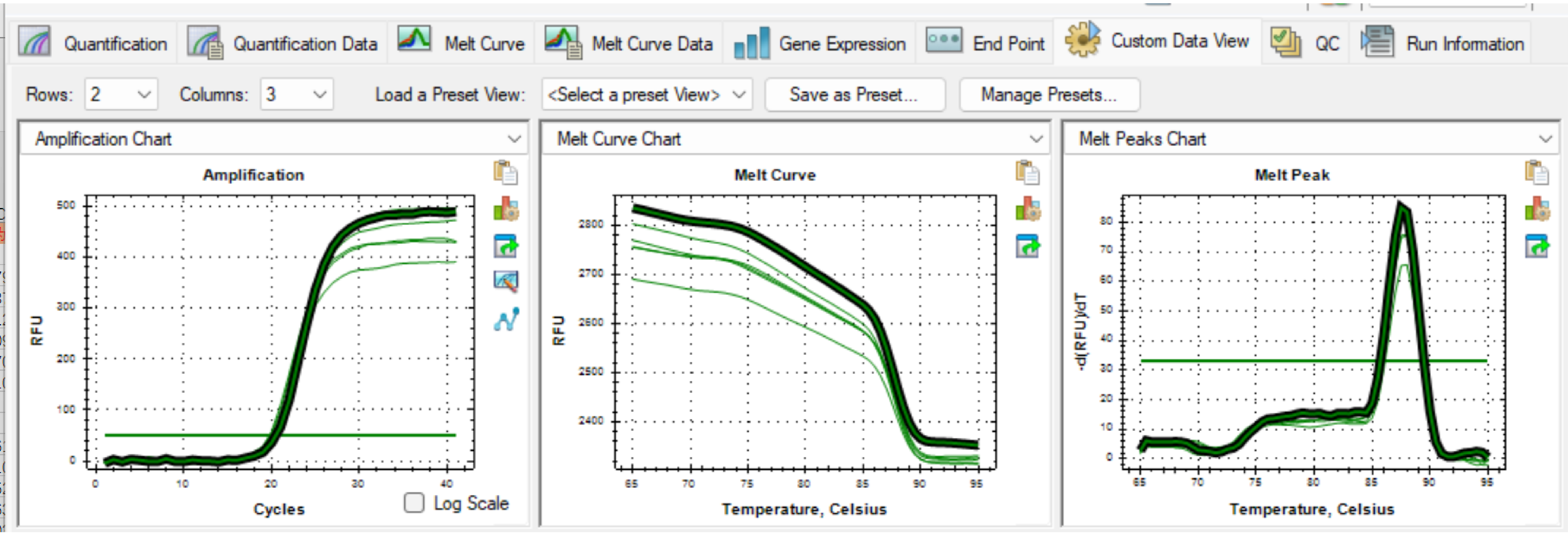

Supplement: Figure S3 The melt curve of each gene in the qRT-PCR assay. [file supplementary_figure_3.pdf]
